# Imaging of large volume subcutaneous deposition using MRI: exploratory clinical study results

**DOI:** 10.1007/s13346-023-01318-7

**Published:** 2023-03-13

**Authors:** Ronald J. Pettis, Wendy D. Woodley, Kevin C. Ossege, Adam Blum, Natasha G. Bolick, Christopher J. Rini

**Affiliations:** 1 BD Technologies & Innovation, 21 Davis Dr., Research Triangle Park, Durham, NC 27709 USA; 2 Kinetic Vision, 10651 Aerohub Blvd., Cincinnati, OH 45215 USA

**Keywords:** Depot localization, Large volume subcutaneous injection, Magnetic resonance imaging, Subcutaneous tissue

## Abstract

Subcutaneous (SC) delivery is a preferred route of administration for biotherapeutics but has predominantly been limited to volumes below 3 mL. With higher volume drug formulations emerging, understanding large volume SC (LVSC) depot localization, dispersion, and impact on the SC environment has become more critical. The aim of this exploratory clinical imaging study was to assess the feasibility of magnetic resonance imaging (MRI) to identify and characterize LVSC injections and their effect on SC tissue as a function of delivery site and volume. Healthy adult subjects received incremental injections of normal saline up to 5 mL total volume in the arm and up to 10 mL in the abdomen and thigh. MRI images were acquired after each incremental SC injection. Post-image analysis was performed to correct imaging artifacts, identify depot tissue location, create 3-dimensional (3D) SC depot rendering, and estimate in vivo bolus volumes and SC tissue distention. LVSC saline depots were readily achieved, imaged using MRI, and quantified via subsequent image reconstructions. Imaging artifacts occurred under some conditions, necessitating corrections applied during image analysis. 3D renderings were created for both the depot alone and in relation to the SC tissue boundaries. LVSC depots remained predominantly within the SC tissue and expanded with increasing injection volume. Depot geometry varied across injection sites and localized physiological structure changes were observed to accommodate LVSC injection volumes. MRI is an effective means to clinically visualize LVSC depots and SC architecture allowing assessment of deposition and dispersion of injected formulations.

Trial Registration: Not applicable for this exploratory clinical imaging study.

**Graphical Abstract:**

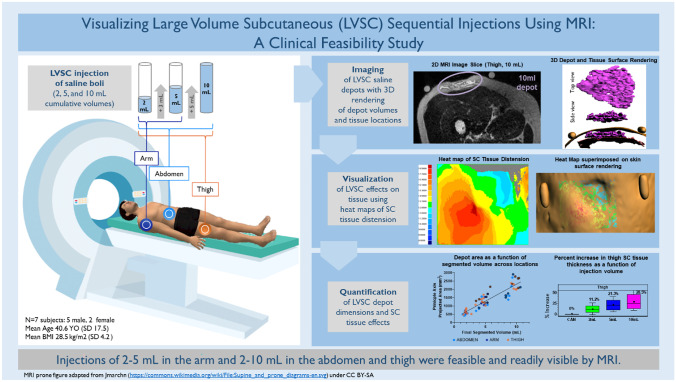

**Supplementary Information:**

The online version contains supplementary material available at 10.1007/s13346-023-01318-7.

## Introduction

Use of biotherapeutics, such as monoclonal antibodies (mAbs), is becoming increasingly prevalent as primary therapy for chronic conditions, including immunologic and inflammatory diseases and cancer. Biotherapeutics require parenteral administration via intravenous (IV), intramuscular (IM), or subcutaneous (SC) injection due to limited oral bioavailability [1]. The SC route is often preferred over IV by patients and healthcare providers due to the convenience of self-administration, the option of non-clinical delivery settings, reduced healthcare costs and time, and improved safety profile [2–6]. These benefits are well-documented, and many biotherapeutics initially approved for IV administration are transitioning to SC delivery [5, 7–9]. However, in contrast to the essentially unrestricted volume limitation of the IV route, SC injection has been predominantly limited to volumes below 3 mL, which is insufficient for many biotherapeutic doses and concentrations [10–12]. Scientific and technological advancements to support large volume SC (LVSC) delivery are emerging, such as the development and commercialization of wearable on-body injectors, larger volume autoinjector devices, and tissue permeation modifiers such as hyaluronidase [10, 12–15]. However, critical knowledge gaps for LVSC injection mechanics and tissue distribution remain, which may be critical for understanding tissue responses to injection, as well as better predicting subsequent physiological uptake and bioavailability.

Located between the intradermal (ID) and IM space, the SC tissue, or hypodermis, is primarily composed of adipose cells and connective tissue and is transited by nerves, blood, and lymphatic capillaries [16]. LVSC administration delivers injectate fluid into the interstitial space with subsequent local tissue permeation and systemic uptake accomplished by blood or lymphatic capillaries [17]. Uptake is impacted by numerous factors including drug molecular weight, which affect lymphatic versus vascular capillary distribution, local capillary density, and interstitial pressure [18–20]. Further, LVSC delivery exhibits varied physical local tissue responses, such as wheal formation and erythema, which are affected by anatomical site differences [13, 14]. Different injection locations (abdomen, thigh, arm) or tissue beds (ID, SC, IM) are also known to impact drug absorption kinetics, presumably due to localized tissue morphology differences [17, 21–25]. A complex relationship exists between physiologic site of LVSC administration, interstitial fluid dynamics, and therapeutic bioavailability [26]. Therefore, understanding SC fluid distribution may be advantageous for optimizing LVSC delivery system design and drug formulations.

Direct visualization of the pattern and location of the SC depot can provide insight into the impact of LVSC delivery on the tissue environment, inform effective delivery system design, and confirm deposition and distribution within the target SC tissue. Macroscopic surface tissue effects (e.g., wheals) are visible by external examination, and pathological effects can be evaluated by invasive tissue biopsy sampling. However, SC injection visualization with quantitative analysis of the underlying depot formation and dispersion requires medical imaging technologies. Clinical and non-clinical studies have assessed the localization of SC depots using ultrasound [13, 14, 27–29], x-ray [30, 31], or computerized tomography (CT) scan [28, 32]. While these imaging modalities have provided qualitative assessment of depot locations, various considerations such as ease of implementation and radiation exposure (e.g., for CT and x-ray fluoroscopy) or precise measurement of depot margins and locations (e.g., for ultrasound) have limited their clinical utilization for quantitative SC depot visualization. In contrast, magnetic resonance imaging (MRI) is capable of high soft tissue spatial resolution and providing localization against anatomical references without the use of either contrast media enhancement or ionizing radiation. Of note, MRI has been used to examine deposition of insulin [33, 34] and dermal fillers [35–37], soft tissue dimensions [38, 39], and injection effects on both SC and IM soft tissues [32, 40, 41]. To the authors’ knowledge, MRI has not been used to directly examine LVSC deposition patterning in humans.

The goal of this exploratory clinical imaging study was to visualize the pattern and location of fluid dispersion in SC tissue as a function of delivery site and volume by MRI. Study objectives included defining an MRI sequence and determining its feasibility for qualifying and quantifying LVSC depot location and establishing methodology and metrics for subsequently rendering three-dimensional (3D) images and characterizing LVSC depots. Incrementally increased injection volumes of 2-, 5-, and 10-mL normal saline were delivered via MRI suite compatible syringe pumps in the upper arm, thigh, and abdomen of healthy human subjects. Imaging was collected at incremental timepoints throughout the injection process, with post-image processing and analysis performed to render and dimensionally characterize the 3D depot. Additionally, tissue dimensions surrounding the delivery site were examined to quantify SC tissue distension resulting from the injection process.

## Materials and methods

### Study subjects

Inclusion criteria included subjects of both genders, 18 to 65 years of age with body mass index (BMI) ≥ 18.5 kg/m^2^. Exclusion criteria included pregnancy, use of anti-platelet/coagulants therapy, bleeding disorder, easy bruising, blood borne infection, skin sensitivities or disorders, fear of injections, analgesic use within 24 h of study injections, and metal-containing implants or patches. Injection sites were visually confirmed to have no gross skin anomalies. Subjects were also excluded if already enrolled in a current clinical study and/or had an employment conflict of interest. As an exploratory study, no population estimates were used to determine subject recruitment or distribution across demographics. Subjects were compensated for their participation. This non-interventional study was approved by the Institutional Review Board (Schulman, now Adverra) and was conducted in accordance with the ethical principles that originate from the Declaration of Helsinki and the Belmont Report. Study conduct complied with US Food and Drug Administration Regulations, applicable state and local regulations, ISO 14155, and the Good Clinical Practice guidelines set forth by the International Council for Harmonisation.

### Study design

This exploratory clinical imaging study was a non-randomized, open-label, single center (Stand Up MRI of Fort Lauderdale, Fort Lauderdale, FL, USA) study designed to evaluate MRI feasibility for LVSC at cumulative injection volumes up to 10 mL administered at various anatomical sites in healthy adult subjects and if successful, depot location and distribution within SC tissue. Subjects were seen twice in clinic: screening (visit 1) to provide written informed consent, demographic information, and inclusion/exclusion eligibility and imaging (visit 2) up to 4 days later.

### Delivery system and injection sites

**Fig. 1:**
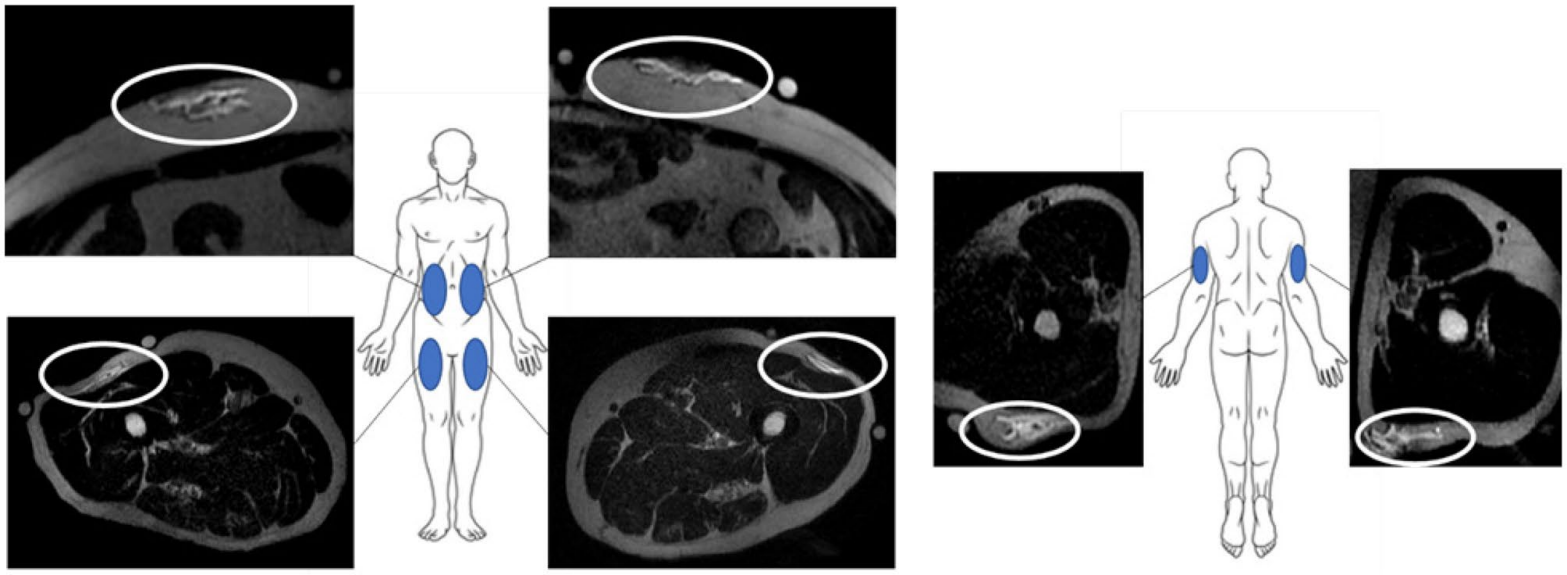
**LVSC injection site locations.** LVSC injection sites include abdomen (upper left), thigh (lower left), and arm (right) with representative MRI axial images showing saline depots (white ovals) at peak injection volume. For abdomen and thigh, incremental delivery was administered up to 10 mL and up to 5 mL in the arm sites. Reference anatomy including bony structures, organs, and soft tissue planes are clearly visible. Permission was obtained to use and modify the human graphic (copyright: Sergey Tkachevto, Dreamstime.com)

All delivery system components were commercially available; no investigational devices or products were used in this study. An MRI suite compatible power injection pump (MEDRAD® MRXperion MR Injection System; Bayer Healthcare, Whippany, NJ, USA) with large volume disposable syringe reservoir (Medstream MS404) was filled with normal saline without contrast enhancement and fitted with a flexible large bore extension set of sufficient length to reach the subject within the MRI unit. Commercial insulin infusion sets (Animas Contact Detach, 6 mm stainless steel 29G cannula or Medtronic Quick-Set™, 6 mm 25G polymer cannula with 27G introduction needle) were connected to the extension set, fully primed, and inserted perpendicular to the skin with bilateral placement to the upper anterolateral thigh and abdomen or right or left upper arm injection sites by trained health care professionals (Fig. 1). Multiple incremental injections of 2-, 3-, and 5-mL were utilized to achieve total cumulative target injection volumes up to 5 mL (2 + 3 mL) in the arm and 10 mL (2 + 3 + 5 mL) in the abdomen and thigh, comprising one injection series at each delivery site. Per protocol, each subject could receive up to 6 injection series total, one in each thigh, arm, and in contralateral sides of the abdomen. A single SC set was used at each site, with a new set primed and placed for each injection series. Total administered volumes per subject were below those used in current routine clinical practices such as SC hydration [42] and SC immunoglobulin therapy for primary immunodeficiency [43, 44]. Confirmation of delivered volume was not a specific study endpoint; however, injection site leakage was not observed for any injection series.

### LVSC injection and deposition imaging

**Fig. 2:**
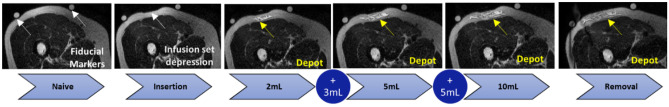
**Imaging and injection sequence:** Representative thigh axial MRI images taken at different volumes during the injection process. Fiducial markers (fish oil capsules) appear as small spheroids floating above the tissue surface (white arrows, naïve scan, left). Minor surface tissue depression associated with catheter set adhesion appears after cannula placement (white arrow, insertion scan, second from left) but disappears after cannula removal (post-removal scan, far right). Incremental depot volumes appear as brighter high contrast regions in the SC space after injection (yellow arrows in 2, 5, 10 mL and post-removal scans)

Fiducial markers (fish oil capsules) were attached on the skin surface using medical tape bracketing the injection sites to enable initial injection site registration (Fig. 2). MRI was performed by an experienced MRI operator using a GE Signa™ HDxt, 3 Tesla Field Strength MRI (GE Healthcare, Chicago, IL, USA). Diffusion images were obtained using a T2-weighted Fast Spin Echo (FSE) MRI sequence with 3 mm slices (1 mm skip) with a resolution of 0.5469 to 0.6641 mm. This imaging sequence was selected from scan optimization cycles on 2 subjects based on a combination of reasonably brief scan cycle time and operator visual inspection of slice image contrast quality at the time of scanning.

Subjects were positioned in the MRI bore for site registration using fiducial markers for localization and pre-cannulation baseline scans, followed by infusion set placement outside the MRI bore. After subject repositioning in the MRI bore, incremental injections of 2-, 3-, and 5-mL normal saline at 0.02 mL/s rate (Fig. 2) were sequentially administered to obtain cumulative injection volumes of 2, 5, or 10 mL, respectively. A single infusion set was used for the entire volumetric series at each injection site, without subject removal from the MRI bore. All images were acquired over a consistent predetermined field area, approximately a 10.8 cm image stack dimension centered on the cannula position. Scanning began immediately upon completion of each incremental injection volume, with the subsequent incremental injection and scan cycle immediately following the prior scan completion. Total injection and scan time was approximately 3–6 min per injection increment. Subjects were further instructed to minimize movement and perform shallow breathing if able during abdominal injection image capture. Scans could be immediately repeated based on operator review of raw images, if necessary, although this was infrequent. All scanning and incremental delivery methods were selected to reduce overall imaging cycle times to limit depot diffusion and motion artifacts between scans. Additional images were obtained post-set removal, which required subject removal from the MRI bore, set removal, and subsequent repositioning in the field. Because this was an exploratory study not powered to a specific endpoint, not all subjects received all possible injection site-volume combinations, and some images in the series were omitted for certain subjects (Table 1; Supplementary Fig. 1, Additional File 1**)**. Neither injection site nor order were randomized as this was not expected to influence outcomes. Injections for each subject were chosen to evaluate as many delivery conditions as possible while considering prior completed deliveries and remaining within the constraints of total enrollment, available injection sites, and accessible imaging suite time.

**Table 1:** Study subject demographic data and injections received

**Subject number**	**Sex**	**Age (yr)**	**Weight (lbs)**	**Height (in)**	**BMI (kg/m**^**2**^**)**	**Method development or analysis**	**Volume administered (mL)**					
							**Abdomen**		**Thigh**		**Arm**	
							**L**	**R**	**L**	**R**	**L**	**R**
001	F	46	200	63	35.4	Method	NA	NA	NA	NA	NA	NA
008	M	18	187	68	28.4	Method	NA	NA	NA	NA	NA	NA
009	M	54	184	70	26.4	Analysis	2	10	10	10	NA	NA
010	F	35	147	66	23.7	Analysis	10	10	10	10	5	NA
011	M	62	178	66	28.7	Analysis	10	10	5	10	NA	5
012	M	18	167	64	24.7	Analysis	5	5	5	5	5	NA
013	M	51	206	67	32.3	Method	10	10	NA	NA	NA	NA
-	**Average**	**40.6**	**181.3**	**66.3**	**28.5**	-	-	-	-	-	-	-
-	**SD**	**17.5**	**20.0**	**2.4**	**4.2**	-	-	-	-	-	-	-

*BMI* body mass index, *yr *years, *F* female, *M* male, *L* left, *R* right, *NA* not available, *SD* standard deviation

### Post-image analysis for depot and tissue surface 3D rendering

**Fig. 3:**
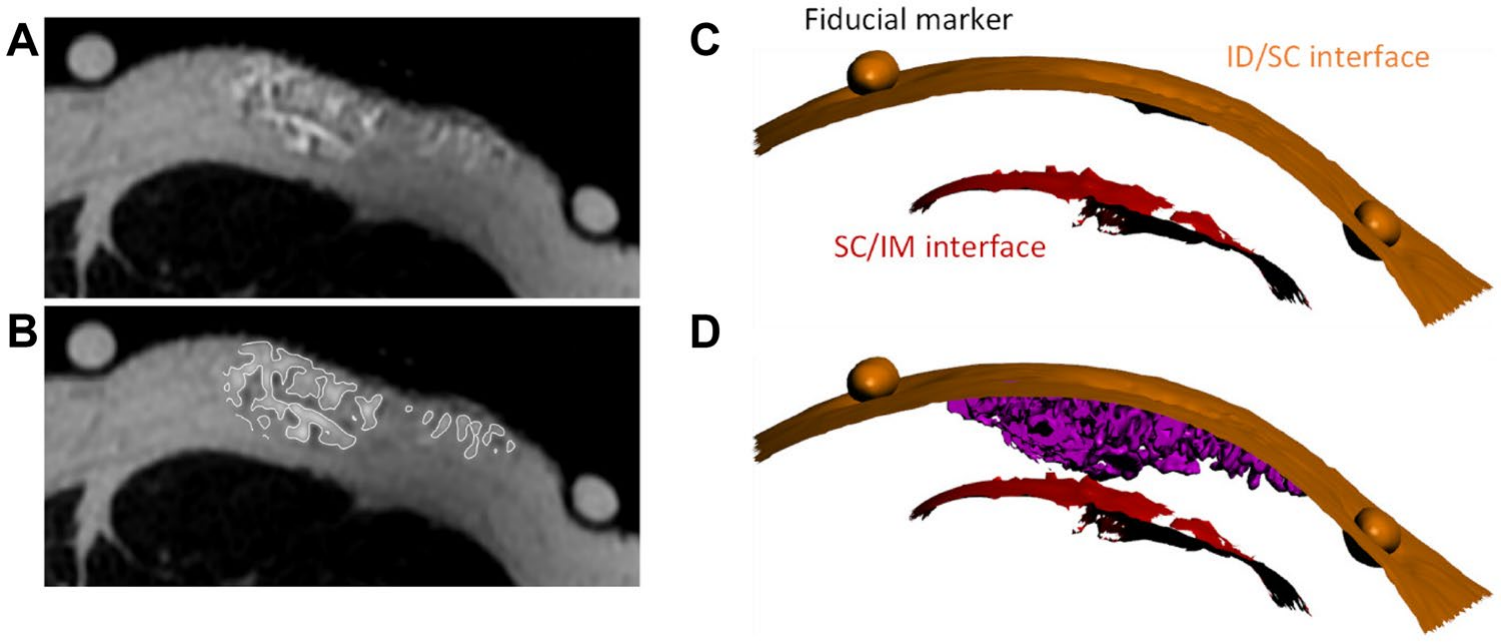
**Depot identification and rendering. A** Representative 10 mL thigh depot greyscale image taken from a single axial slice (Subject 010, left thigh), including fiducial markers. **B** Same representative axial slice showing outline of depot region of interest segmented from the injection site based on brighter grayscale voxel values vs. surrounding tissue. **C** 3D surface renderings of the ID/SC and SC/IM boundaries including fiducial markers. **D** Segmented 3D depot surface rendering showing relative location of the rendered 3D depot within the subcutaneous tissue bed

Post-image analysis was performed by Kinetic Vision (Cincinnati, OH, USA) to identify depot tissue location (ID, SC, IM), create 3D depot renderings, and estimate in vivo bolus volumes and SC tissue distention. The DICOM® stacks of the MRI scans were imported into VGSTUDIO MAX™ software (Volume Graphics; Heidelberg, Germany) for segmentation and initial measurements. The regions surrounding the depot and injection site were extracted from the full scan to focus analysis on only the region of interest. The surface determination function was then used to segment and create a 3D surface rendering of the depot based on the grayscale values of the extracted region scan histogram (Fig. 3A–B). The histogram is a count of voxels (3D pixels) based on their corresponding grayscale values with grayscale brightness values correlating to different substrate materials, dependent on the type of MRI sequencing used. In the MRI sequence utilized, the depot is a brighter intensity than the surrounding tissue. Using additional region-specific surface determinations, 3D surface renderings of the ID/SC and SC/IM boundaries were also generated (Fig. 3C–D).

Once the 3D surface renderings were generated for the depot, ID/SC, and SC/IM tissue boundaries, measurements and visualizations were taken from each set of scans. The volumes of the segmented depots were recorded and compared to the target injection volumes. Automation batch processing was developed to measure the minimum 3D distance of the depot to both the ID/SC and SC/IM tissue boundaries using the Geomagic® Control X™ software (3D Systems, Rock Hill, SC, USA). This same software was used to measure the 3D distance between the ID/SC and SC/IM tissue boundaries directly. To generate additional visualizations and overlays of the various 3D surface renderings, scans from the same subject and injection site series were aligned to one another using the post-injection scan as a reference, with the additional surfaces being aligned using a least-squares fit of the ID and fiducial marker surfaces in the region of the injection site.

### Imaging artifacts and resolution

**Fig. 4:**
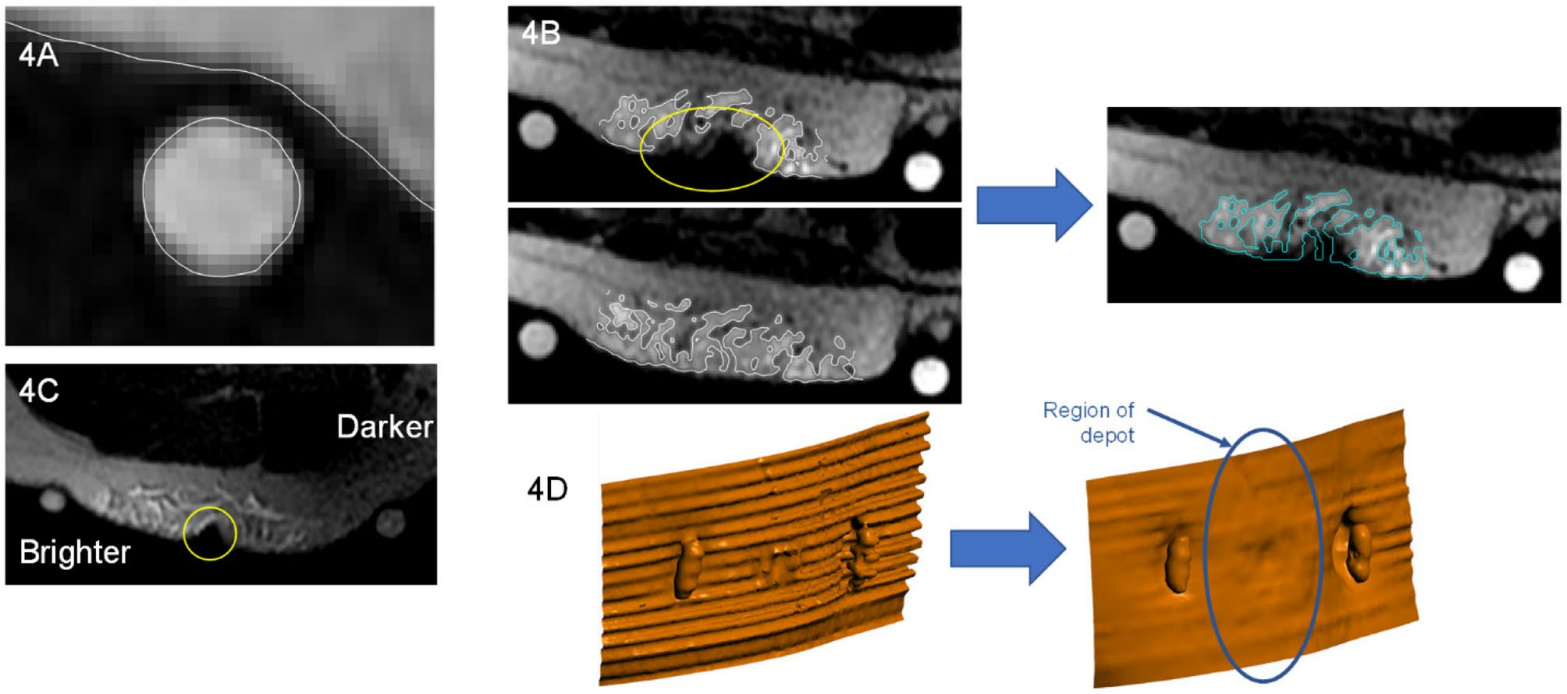
**Image artifacts and reconstruction methods.** **A **Fish oil contents of the fiducial markers and the ID/SC interface are highlighted (white lines). The dermis was not specifically visible in the imaging sequence, making direct ID thickness and depot position measurements impossible. The average dermal gap was reduced by the average thickness of the fiducial marker capsule shell to calculate dermis thickness and relative depot locations. **B** Representative abdominal image showing image voids due to steel needle immediately around the injection site (yellow oval) which was reconstructed by referencing the infusion scan postneedle removal (bottom), creating a backfilled depot (right). **C** Representative thigh axial image demonstrating brighter (left side) and darker (right side) attenuation variability across the same depot scan region, which may have impacted final depot segmentation. Partial needle attenuation is also shown (yellow circle). **D** Representative abdominal image demonstrating a stair-step artifact (left) due to breathing, which was noted in all abdominal scans and affected both tissue layer (skin surface shown) and depot renderings. Algorithmic realignment of step slices smoothed the artifact immediately around the depot region (blue oval) allowing tissue distance and depot measurements

Four image artifacts were identified during the 3D depot rendering (Fig. 4) with various corrections applied as follows.

First, because of the sequence used, the dermis (skin surface and ID tissue) was not specifically visible in the MRI scans (Fig. **4**A), appearing as a gap between fiducial markers and underlying ID/SC interface. The visible upper SC boundary was used as a surrogate for the lower dermal surface for 3D renderings. Dermis thickness was estimated by subtracting the average fish oil capsule wall thickness (determined by empirical caliper measurements) from the gap width between the imaged fish oil inside the capsule and the SC tissue.

Second, MRI scans of injections administered via the 29GA metal cannula displayed field interference during imaging, leaving an imaging void around the cannula insertion site that obscured immediate portions of the surrounding tissue and depot (Fig. 4B). The depot volume that was obscured in the affected scans was backfilled by interpolating the depot margins from the post catheter removal scan where no metal cannula was present. Because all voids occurred in the region directly adjacent to the cannula with the surrounding depot present in all injections, using the post catheter removal scan seemed reasonable to approximate the depot shape and volume of obscured regions.

Third, in some instances within scans of individual depots, variable grayscale attenuation was observed across the surrounding regions of interest, with depots spanning between brighter and darker regions of the scan (Fig. 4C). The variability in grayscale brightness within a single scan made voxel selection and segmentation more difficult with some resulting loss of depot detail. Segmentation methods were kept as consistent as possible but could have led to higher errors for depot volume estimation in such instances.

Fourth, despite efforts to minimize motion artifacts, 3D reconstructed abdominal scans contained a breathing artifact, creating a stair-step artifact between the MRI slices (Fig. 4D). This artifact can be seen across both the depot volume and reconstructed tissue surfaces, which interfered with measuring depot distance between the ID/SC and SC/IM tissue interfaces. A custom automation script was developed to perform basic slice-to-slice realignment of the ID/SC and SC/IM boundary surfaces within the MRI scan. This slice-to-slice alignment smoothed the ID/SC and SC/IM surfaces allowing for more accurate distance measurements of the tissue boundaries to the depot and one another.

### Evaluation of depot dimensions

After image artifact adjustment, if required, the volume and approximate depot locations were calculated. Depot depth from dermis was based on the distance of the depot top from the ID/SC boundary surface (as a surrogate for the lower ID). Depot depth from the skin surface was calculated as depth from ID/SC interface plus the mean calculated dermal thickness noted in image artifact resolution. Depot depth to the muscle surface was extracted directly from the rendered reconstructions. A separate alignment was performed for dimensional information of the depot itself. The rendered depot volumes were oriented in the X–Y plane parallel to the skin surface using Geomagic Wrap® (Artec 3D, 20 rue des Peupliers, L-2328, Luxembourg) software to generate orthographic two-dimensional (2D) depot surface area projections and identify associated depot principal axes dimensions (widest and secondary dimension parallel to skin surface). These dimensions were subsequently examined as a function of depot volume and location. Basic descriptive statistics (mean, median, standard deviation [SD], range) were applied to examine trends across LVSC injection dimensions (Supplementary Tables 1 and 2, Additional File 3).

### Effect of depot on surrounding SC tissue

To measure local tissue expansion across increasing deposition volumes, the SC tissue thickness was quantified in Geomagic® Control-X™ (Artec 3D, 20 rue des Peupliers, L-2328, Luxembourg) by mapping the 3D distance between the ID/SC and SC/IM boundaries, which were both discretely visible on scans. ID and IM surfaces surrounding the depots were rendered after all image artifact adjustments. The point where the depot was closest to the IM surface in the largest LVSC injection sequence (10 mL for thigh and abdomen, 5 mL for arm) was determined for each subject and location. This point was interpolated to the IM surfaces for all other respective injection sequences (e.g., pre-injection, 2 mL, 5 mL, post-injection). The minimum projected 3D distance from this IM point to the ID surface was then measured to obtain a single SC tissue thickness value at the same location for each individual site and injection sequence. SC tissue thickness before injection and after sequential LVSC injections is represented as a skin surface color heat map for each individual site and injection sequence to show the underlying tissue expansion for volume accommodation.

### Statistical methods

As an exploratory study, this study was not powered to detect specific differences associated with site, volume, or demographic variables. Post hoc summary descriptive statistics (number of observations, mean, standard error of the mean, SD, minimum and maximum) were calculated and presented for all quantitative responses. Such quantitative aspects should be considered in light of artifacts and image adjustments described previously.

## Results

### Demographics

Seven healthy male (*n* = 5) and female (*n* = 2) subjects with a mean age of 40.6 years (SD 17.5 years) and mean BMI of 28.5 kg/m^2^ (SD 4.2 kg/m^2^) were enrolled in this study (Table 1). Two subjects (Subjects 001 and 008) were imaged to refine the MRI sequence method and their injections were not used for analysis. An additional subject (Subject 013) received a single injection of 10 mL into the right and left abdomen that was imaged at 10‑min intervals through 80 min post-injection for future method development (Table 1). The remaining 4 subjects (Subjects 009 to 012) received incremental injections from 2 to 10 mL cumulative (site dependent) per the injection sequence displayed in Fig. 2 with accompanying MRI images taken.

### Visualization of depots using MRI

An objective of this study was to define an MRI imaging sequence and determine the feasibility of that sequence for qualifying and quantifying LVSC depot location and metrics.

MRI images were collected pre-injection, post-cannula insertion, and incrementally following pump-mediated saline injections. The T2-weighted FSE MRI sequence with 3 mm slices (1 mm skip) was selected from various sequences attempted during initial sequence optimization on 2 subjects based on trained operator visual inspection of raw image data, albeit with certain artifacts as noted in the “Imaging artifacts and resolution” section.

**Fig. 5:**
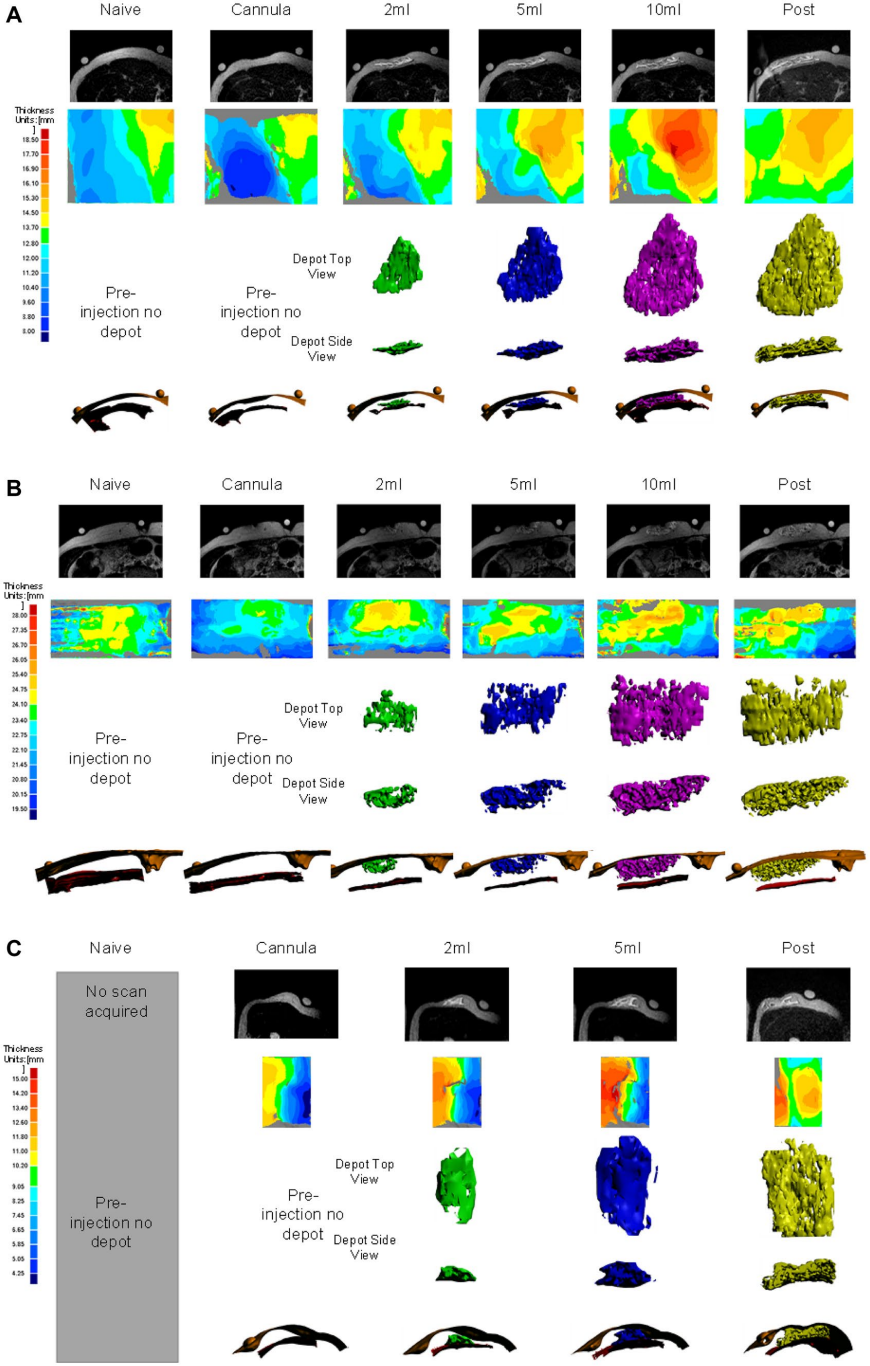
**Representative MR slices, depot and tissue reconstructions, and SC tissue thickness heatmaps from LVSC thigh (A), abdomen (B) and arm (C) injection sites**: (top rows) Each image series ranges from naïve tissue/catheter placement through maximum injection volume (left to right) up to 10 mL cumulative injection in thigh and abdomen and 5 mL in the arm. (Second rows) SC thickness heat maps of the area around the injection site demonstrate increasing thickness with volume (dark blue increasing to yellow, orange, and dark red sequentially). SC thickness heat map scales vary with each specific tissue locale. (Lower 3 rows) Reconstructed 3D images demonstrating top and side views of the 2** mL** (green), 5 mL (blue), 10 mL (magenta) depots and post-set removal (yellow) depots alone. The 3D depot images within the ID/SC interface and SC/IM interface give context to depot location and topology within the tissue. Depot dimensions are not shown to scale between rows or between injection sites but meant to show relative depot shapes and locations; depot dimensions for each example site are shown to scale across columns of increasing volume

Using this sequence without addition of contrast media, MRI was able to readily visualize increasing injection volumes from 2 to 10 mL at common LVSC delivery sites. Representative image sequences across various sites and volumes including 2D slices, rendered depots and tissue planes, and SC tissue expansion heatmaps are presented in Fig. 5. Comprehensive images and depot/tissue reconstructions from this study in similar format can be found in the Supplementary information, Additional File 1.

### Generation of 3D in situ renderings of the depots

Another study objective was to determine if MRI data could be compiled into comprehensive 3D depot renderings of fluid depot in tissue. 3D renderings of the SC depots alone and in relation to the ID/SC and SC/IM interfaces were created for each injection series from stacked parallel axial MRI scanned slices with adjustments for observed image artifacts (Fig. 5; Supplementary Fig. 1, Additional File 1). Variable depot shape and depth was observed across all sites and volumes, with non-uniform SC spreading in all planes. Local tissue and site appeared to impact dispersion based on direct observation of scan depots and surrounding tissues, and in many cases appeared to cause the SC tissue to expand and conform to the local tissue topography. Likewise, depots had numerous observed surface gaps, voids, and non-spheroidal shapes indicating that injected fluid was likely following a path of least resistance through the SC matrix.

**Fig. 6:**
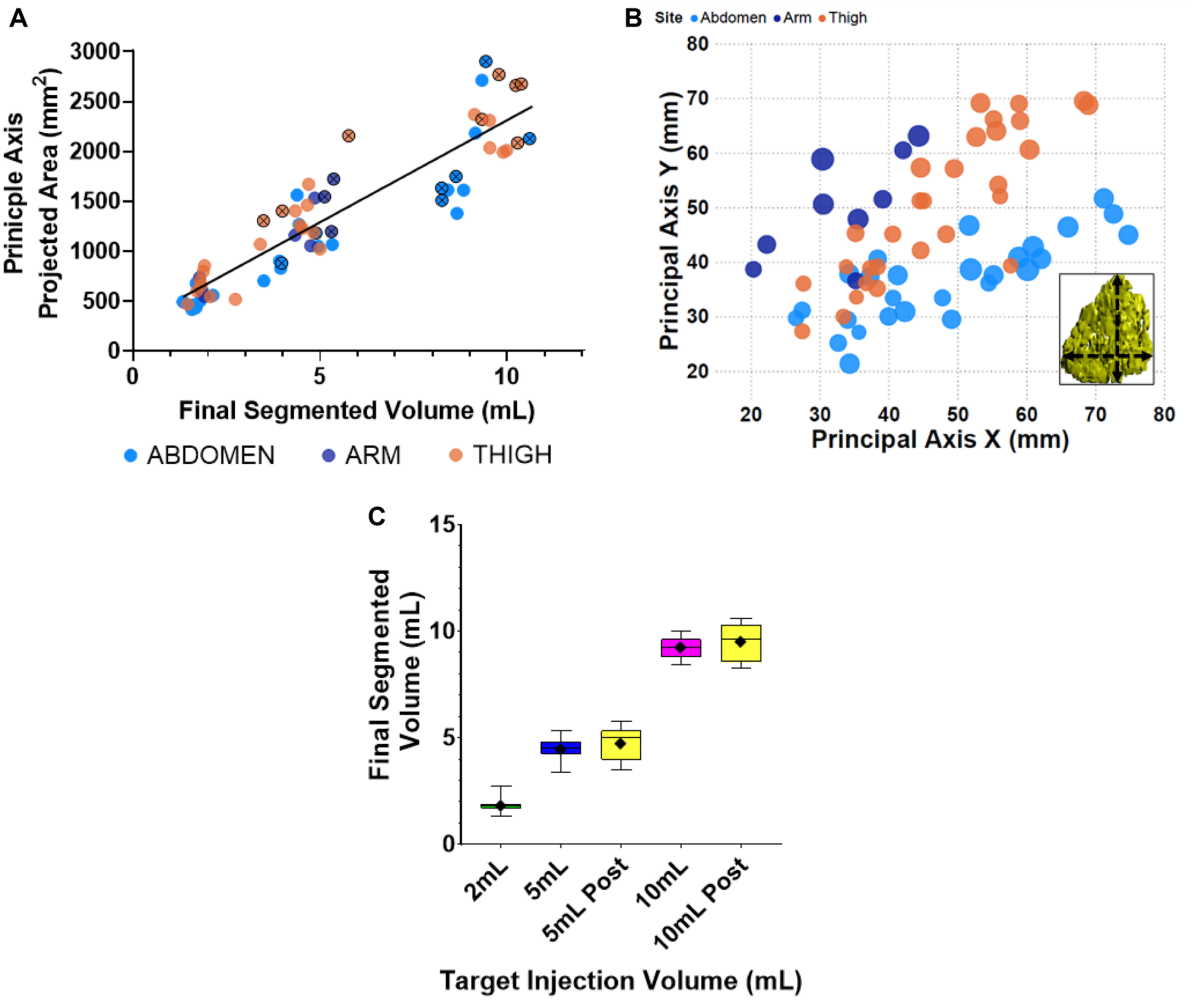
**Depot dimensions as a function of injection conditions**. **A **Depot areas from 2D orthogonal projections for all depots, plotted per injection site vs. final segmented depot volume. **B** Principal X–Y depot axis dimensions (inset) across all volumes as a function of injection site. Relative Z dimension is represented by circle diameter. X–Y depot spreading trends appear to be injection site–dependent. **C** Boxplot of final segmented depot volume vs. target injection volume across all depots regardless of site (mean indicated by diamond symbol, median by line, and SD by error bars)

As expected, projected 2D depot surface areas increased as a function of final corrected segmented volume regardless of site (Fig. 6A). Interestingly, the principal X–Y dimensional relationship across volumes also exhibited differences as a function of injection site location (Fig. 6B), potentially due to known differences in localized tissue morphology such as underlying muscle, bony or organ structures or site-specific differences in SC tissue thickness [45, 46]. Additionally, the final calculated rendered depot volumes were similar to the target total injectate volumes (Fig. 6C). Overall, the MRI 3D renderings provide effective visualization of irregular depot expansion and increasing local distribution with increasing injectate volume.

**Fig. 7:**
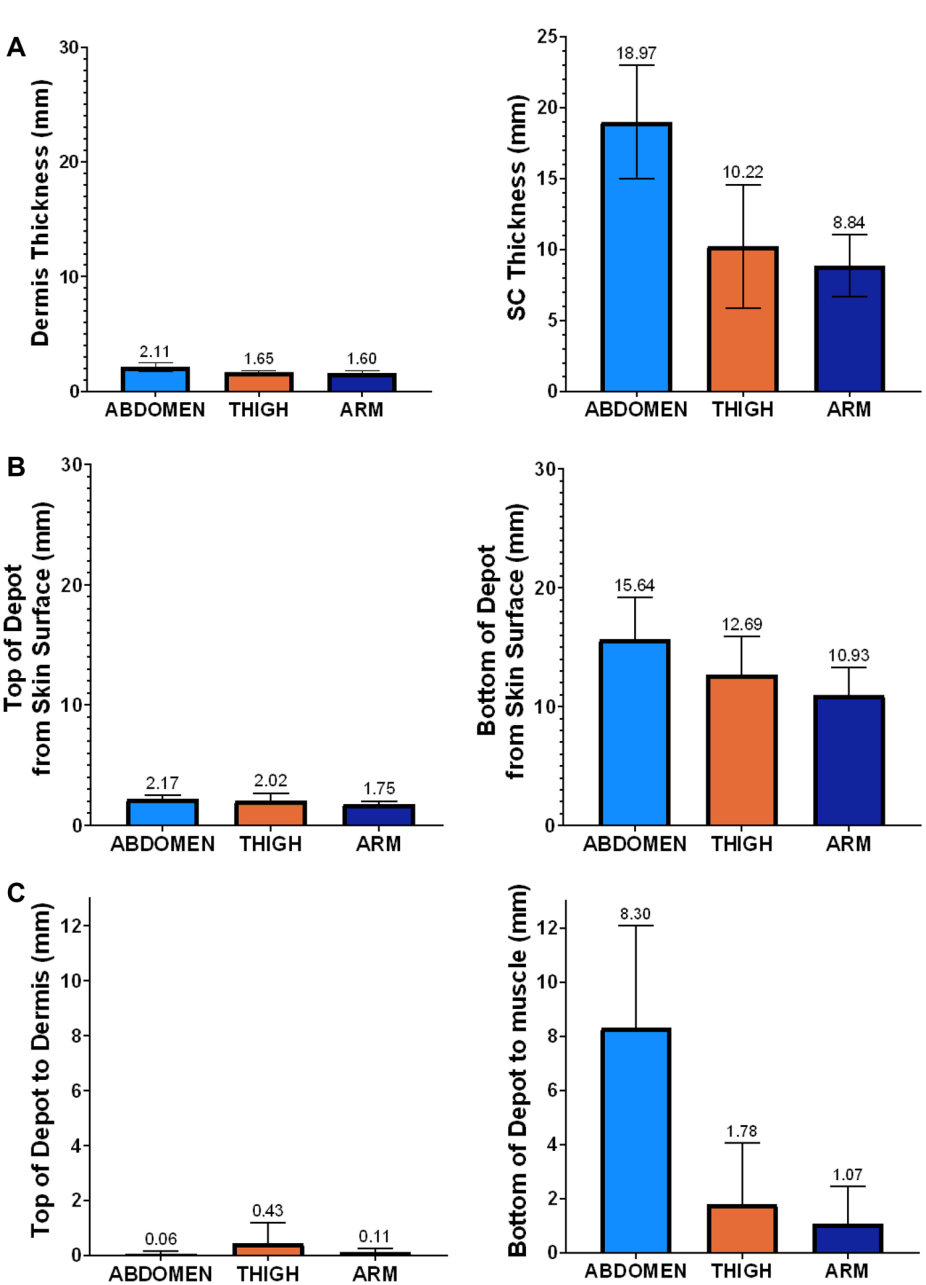
**Tissue characteristics and depot locations as a function of site averaged across all volumes (mean ± SD): A** Dermal and SC tissue thickness. **B** Approximate SC location of LVSC depots relative to skin surface. **C** Approximate location of LVSC depots relative to ID and muscle in the SC tissue

Depot renderings including the ID/SC and SC/IM tissue boundaries appear to demonstrate predominantly SC deposition (Fig. 5, Supplementary Fig. 1, Additional File 1), though exclusive SC deposition cannot be explicitly confirmed due to image artifacts as described in the “Imaging artifacts and resolution” section. Mean tissue dimensions (Fig. 7A) and relative depot locations within the tissues (Fig. 7B–C) were calculated from the image renderings. Regardless of LVSC injection volume, depot top borders (Fig. 7B) routinely appeared just at or below the ID/SC boundary. This is consistent with prior preclinical and published clinical experience for 5- and 10-mL deliveries measured using other imaging modalities, including fluoroscopy and ultrasound [13]. With increasing volume, depots were closer to the underlying muscle layer (Supplementary Fig. 2, Additional File 2), with differences most obvious for abdominal injections. Similarly, the abdominal lower depot borders displayed an increased distance from muscle layer (Fig. 7C) compared to thigh and arm, presumably owing to increased overall SC tissue thickness at this site.

### Measurement of SC tissue thickness with increasing depot volume

**Fig. 8:**
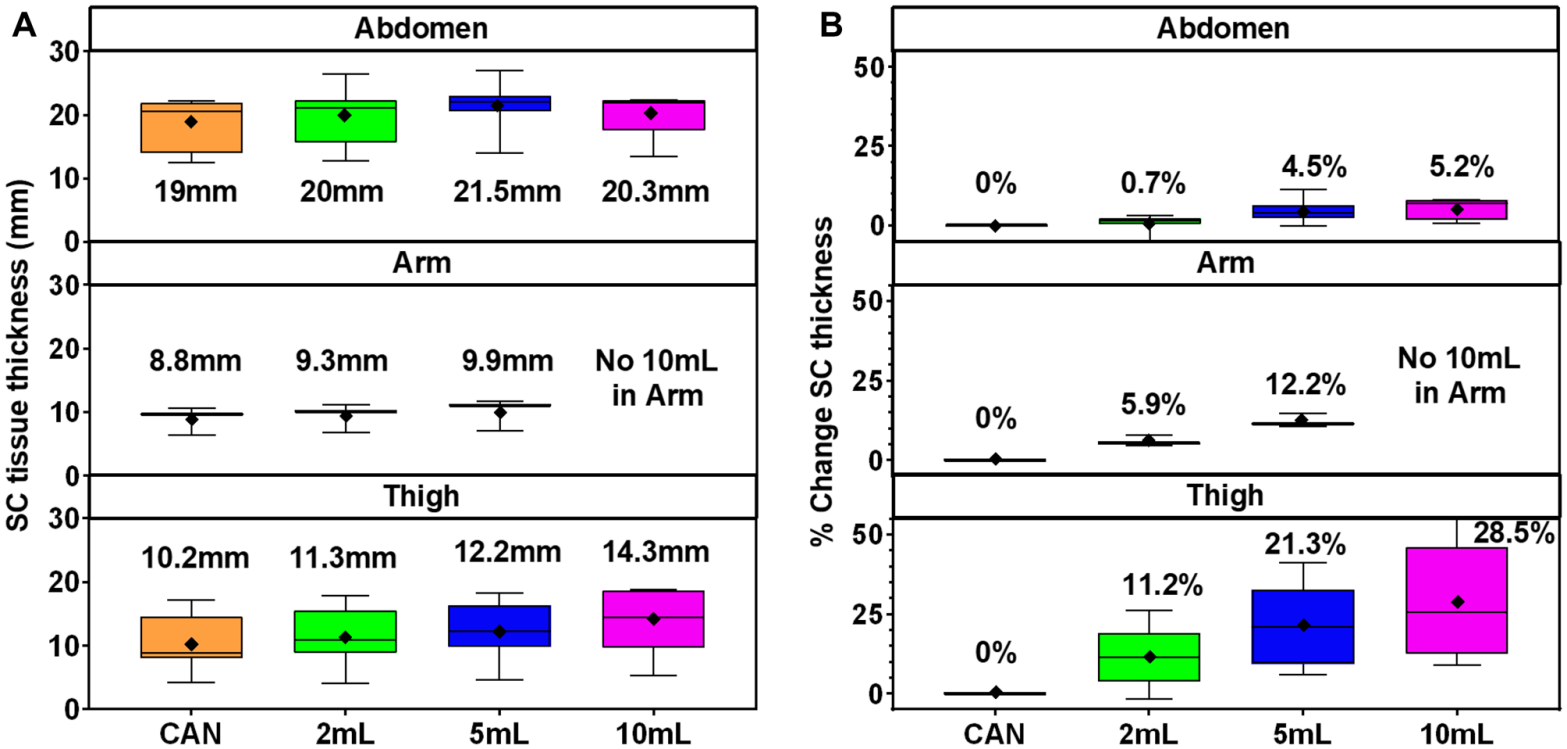
**Effect of injection volume on SC tissue:** **A** SC thickness (mm) was measured as the distance between the visible ID/SC to SC/IM tissue interfaces using the deepest depot point as reference across images. Boxplot with the average (diamonds), median (line) and min to max error bars of SC tissue thickness per injection site (abdomen, arm, and thigh) and time point for injection volume sequences. **B** Percent change in SC thickness compared to cannula placement per injection site and time point for the injection volume sequences. Pre-cannulation and post-cannula removal measurements not included as these may not show the potential tissue compression due to the catheter set

The SC tissue thickness was measured and compared across each injection sequence and site (Fig. 8A). For each injection site, the SC tissue thickness (distance between the ID/SC and SC/IM boundaries) increased with each cumulative injection (Fig. 8A). In the abdomen, the mean SC thickness increased from 19.0 mm (SD 4.0) at cannula placement to 20.3 mm (SD 3.8) after 10 mL cumulative deposition (Supplementary Table 3, Additional File 3). Similarly, the thigh SC thickness increased from 10.2 mm (SD 4.3) to 14.3 mm (SD 5.5) at 10 mL total injection. The arm SC thickness averaged 8.8 mm (SD 2.2) at cannula placement and increased to 9.9 mm (SD 2.5) following 5 mL cumulative injection volume.

To determine the percent change in SC thickness, the measured values for each injection site and volume were compared to those at cannula placement. As summarized in Fig. 8B, SC expansion to accommodate increasing deposition volumes was observed across all injection sites though amount of expansion varied. The largest absolute and percent change in SC tissue thickness was observed in the thigh with a mean increase of 11.2% (SD 9.2), 21.3% (SD 12.8), and 28.5% (SD 18.9) at 2, 5, and 10 mL, respectively (Fig. 8B, Supplementary Table 4, Additional File 3). Similar but more moderate changes were observed in the arm where the SC expanded by a mean of 5.9% (SD 1.6) at 2 mL and 12.2% (SD 2.3) at 5 mL. Abdominal changes were comparatively minor with a mean of 0.7% (SD 2.8), 4.5% (SD 3.8), and 5.2% (SD 3.3) at 2, 5, and 10 mL, respectively. These results demonstrate that depots are locally distending tissues around the local injection area. The observed variability in tissue expansion may reflect site-specific SC tissue layer thickness, topography, and extracellular matrix (ECM) properties.

Subsequently, heat maps were generated to help visualize areas most affected by SC tissue thickness changes in relation to the overlying skin surface. Heat maps represent a top-down view of SC thickness near the needle insertion site from the upper SC boundary (ID/SC interface) inward towards the bottom SC boundary (SC/IM interface). Representative images are displayed in Supplementary Fig. 3 (Additional File 2) where blue is the shallowest SC thickness and colors warm towards dark red as the SC thickness increases. In the various heat map series, the initial shallowest SC thickness often appears beneath the circular adhesive pad of the adhered infusion set where slight tissue compression may occur after cannula placement. However, with increasing injection volume, the local SC tissue expands in thickness to accommodate the deposition as noted above. Heat maps also highlight that such areas of increasing tissue thickness may occur anywhere near the injection site, not just directly below the cannula, a finding also visible in the 2D depot axial image slices and 3D renderings. SC tissue thickness data are also portrayed by histogram, which depicts the percent frequency of SC thickness at the selected site region of interest across each injection volume (Supplementary Fig. 3, Additional File 2). The histogram emphasizes the shift to increased SC tissue thickness across the depot region as the injectate distributes through the ECM.

## Discussion

SC delivery of biotherapeutics offers various advantages over traditional IV infusions [2–6] and is becoming increasingly prevalent for the treatment of numerous chronic conditions. To accommodate SC administration, numerous higher volume drug formulations and delivery systems are either commercialized or under development, yet a firm understanding of LVSC delivery impact on the SC tissue environment remains unclear [13, 14]. Such understanding requires imaging methodologies to assess the localization and distribution of LVSC deposition. In this exploratory clinical imaging study, we have demonstrated that MRI is an effective means to qualify and quantify in situ LVSC deposition and its corresponding mechanical effects on the SC space in healthy human subjects, without the need for contrast enhancement or ionizing radiation.

Saline depots of up to 5 mL in the arm and up to 10 mL in the abdomen and thigh were technically feasible and readily visible by MRI. The in situ depots were observed to occur predominantly within the SC tissue and were non-uniform in nature. From the MRI image series, 3D renderings were created for both the depot alone and in relation to the upper ID/SC and lower SC/IM interfaces that define the SC tissue boundaries. The 3D renderings enabled assessment of in situ depot locations and dimensions. Similarly, localized physiological structure changes were readily observable with visualization of SC expansion patterns within the tissue at increasing injection volumes. Depot geometries varied across injection sites, and 3D tissue spreading appeared to be influenced by local SC structures and tissue environment emphasizing their potential impact on fluid dispersion. The converse impact of LVSC deposition on SC structure was also documented by increases in absolute SC tissue thickness measurements with increasing incremental injection volume. SC distension was also non-uniformly distributed around the injection site.

The subcutis is a complex structure composed of fatty lobules, adipocytes, and other cellular components ordered within a complex non-uniform 3D ECM network composed of collagen, elastin, and glycosaminoglycans, bathed in extracellular fluid and perfused with unevenly distributed blood and lymphatic capillaries and vessels [20, 47]. Further within the overall subcutis, these lobular structures vary with tissue depth and ECM proximity to surrounding dermal and muscular fascia, and with gender, BMI, and age factors [20]. The ECM as the principal determinant of subcutis mechanical properties also acts as the primary impediment to dispersion due to a combination of steric, electrostatic, and specific binding interactions [20, 25]. Therefore, the depot non-uniformity and site variability observed in this study may be readily attributable to the localized tissue environment at the various sites including structural differences due to a thinner SC layer in the thigh and arm compared to the thicker SC tissue in the abdomen, underlying physiological structures, or external positioning influences. These findings correlate well to other imaging and pathological examinations of SC delivery albeit at the significantly lower injection volumes for insulin administration, which noted similar anisotropic dispersion across a path of least resistance for fluid flow following interstitial pathways and around in situ localized tissue structures [48, 49]. This variability in depot characteristics and the numerous impacting factors remain of critical importance as tissue dispersion and the ability to access capillary beds remain essential for subsequent uptake and absorption.

The outcomes for other critical aspects of interest for LVSC injection must also be viewed within the context of study limitations. Sequential cumulative injections to achieve a larger total injection volume is not routine clinical therapy. In this exploratory study, this incremental delivery strategy provided an opportunity to increase imaging replicates across multiple volumes at common SC injection sites. This approach also provided an ability to examine how depots expand as a function of increasing volume. Likewise, although precise measurements of the depot area and volume were affected by image void space filling and smoothing, these were sufficient to show distinct dimensional relationships to injectate volume between anatomical sites. Volumetrically, the rendered image depots were similar to or slightly below target administration volumes. This was counterintuitive considering that fluid dispersion occurs through the interstitial space with a required exclusion volume for cell and tissue components, therefore a larger volumetric area to accommodate the depots might have been predicted. This reduced reconstructed depot volume has precedence from insulin deposition patterning [49] and may be explained by potential imaging sensitivity limitations especially near the depot perimeter where fluid diffusion is occurring. Additionally, the saline placebo utilized in this study likely represents a best-case scenario for rapid dispersion due to its low viscosity and molecular size. Future work should aim to examine LVSC injections with MRI across a range of drug formulations and viscosities, as the impact of chemical and physical property interactions with injection site physiology remains poorly understood [26].

Because the dermal layer was not explicitly visible with this imaging sequence, depots cannot be claimed to be exclusively within the SC tissue boundaries. However, it was noted with approximate depth reconstruction and with images of the upper SC boundary layer, that LVSC depots often fill to just below the calculated dermal boundary. Further, although the dermis and SC layers are physiologically distinct, no discrete structure like the basement membrane between the epidermis and dermis segregates these 2 layers [50]. Therefore, some minor fluid infiltration into the lower dermis could occur with large volume SC administration, although this could not be expressly imaged in this study.

Future refinement of the MRI sequence, delivery system, and procedural logistics may help to reduce imaging artifacts and improve deposition location accuracy and depot dimensional precision. Overall, MRI provided high resolution for depot visibility within the broader anatomical structure, which enabled the generation of 3D in situ depot renderings of LVSC injections in the abdomen, arm, and thigh.

## Conclusions

In conclusion, injections of up to 5 mL in the arm and up to 10 mL in the abdomen and thigh were feasible and visible by MRI. MRI is an effective means to clinically visualize LVSC depots and SC architecture, allowing assessment of dispersion of injected formulations in near real time. In addition, MRI of LVSC depots can provide valuable information regarding injection depth and consistency to support and inform future LVSC delivery, device development, and refinement of predictive delivery models. Further evaluation of dispersion over time may provide unique insight to physiologically relevant uptake as a function of formulation characteristics, injection site tissue structure environment, and delivery system. Additional optimization of the delivery system and MRI sequence may reduce imaging artifacts and provide improved characterization of LVSC boluses and resultant effects on surrounding SC tissue.

## Supplementary Information

Below is the link to the electronic supplementary material.

Supplementary file1 (PDF 4.92 KB)

Supplementary file2 (PDF 159 KB)

Supplementary file3 (PDF 90.2 KB)

## Data Availability

Due to its proprietary nature, additional supporting data cannot be made openly available; however, the majority of data captured and analyzed are represented in supplemental figures and tables.
