# Supplementary material for: Imaging of large volume subcutaneous deposition using MRI: exploratory clinical study results
Source: Drug Deliv Transl Res. 2023 Mar 13;13(9):2353–66. doi: 10.1007/s13346-023-01318-7 (PMC10382358; doi:10.1007/s13346-023-01318-7)

Supplemental Figure 1: All MR images, depot and tissue reconstructions, and SC tissue thickness heatmaps from LVSC thigh, abdomen, and arm injection sites. (Top row) Each image series ranges from naïve tissue/catheter placement through maximum injection volume (left to right) up to 10 mL cumulative injection in thigh and abdomen and 5 mL in the arm. (Second row) SC thickness heat maps of the area around the injection site demonstrate increasing thickness with volume (dark blue increasing to yellow, orange, and dark red sequentially; scales vary with each specific tissue locale). (Third and fourth rows) Reconstructed 3D images demonstrating top and side views of the 2 (green), 5 (blue), 10 (magenta) mL and post set removal (yellow) depots alone. (Bottom row) The 3D depot images within the ID/SC interface and SC/IM interface give context to depot location and topology within the tissue. Depot dimensions are not shown to scale between rows or between injection sites but meant to show relative depot shapes and locations; depot dimensions for each example site are shown to scale across columns of increasing volume.

009 Abdomen Left

Naive	Cannula	2ml	5ml	10ml	Post

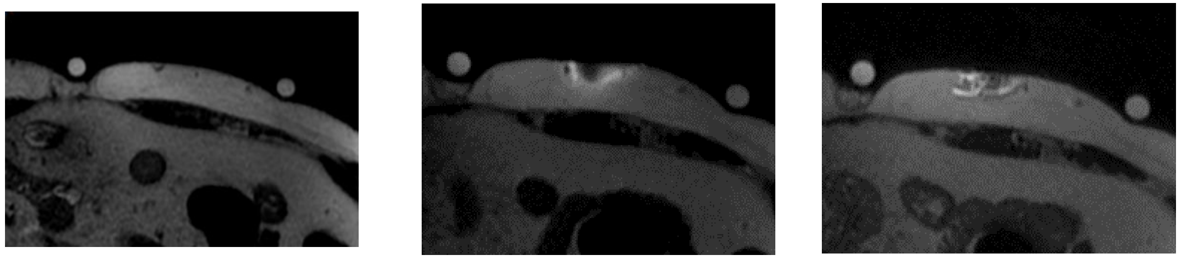

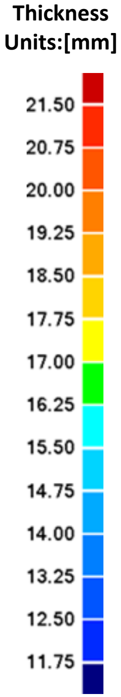

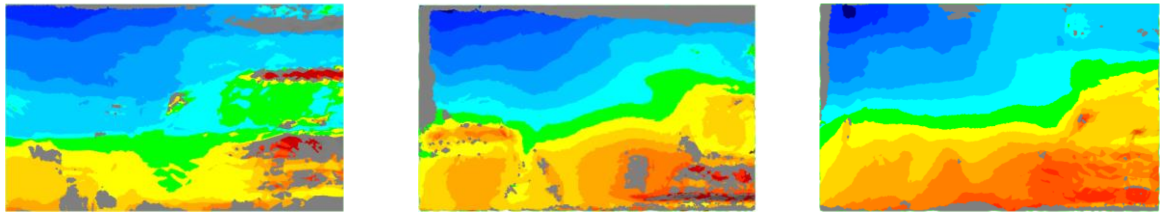

Naive: Pre-injection no depot

Cannula: Pre-injection no depot

Depot Top View

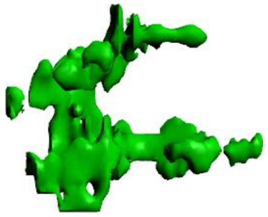

Depot Side View

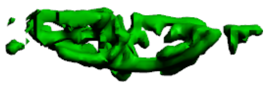

5ml: No scan acquired

10ml: No scan acquired

Post: No scan acquired

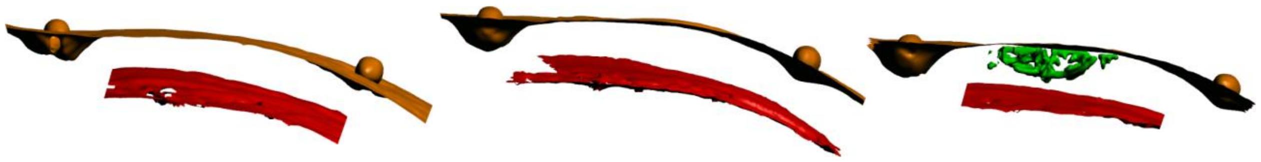

009 Abdomen Right

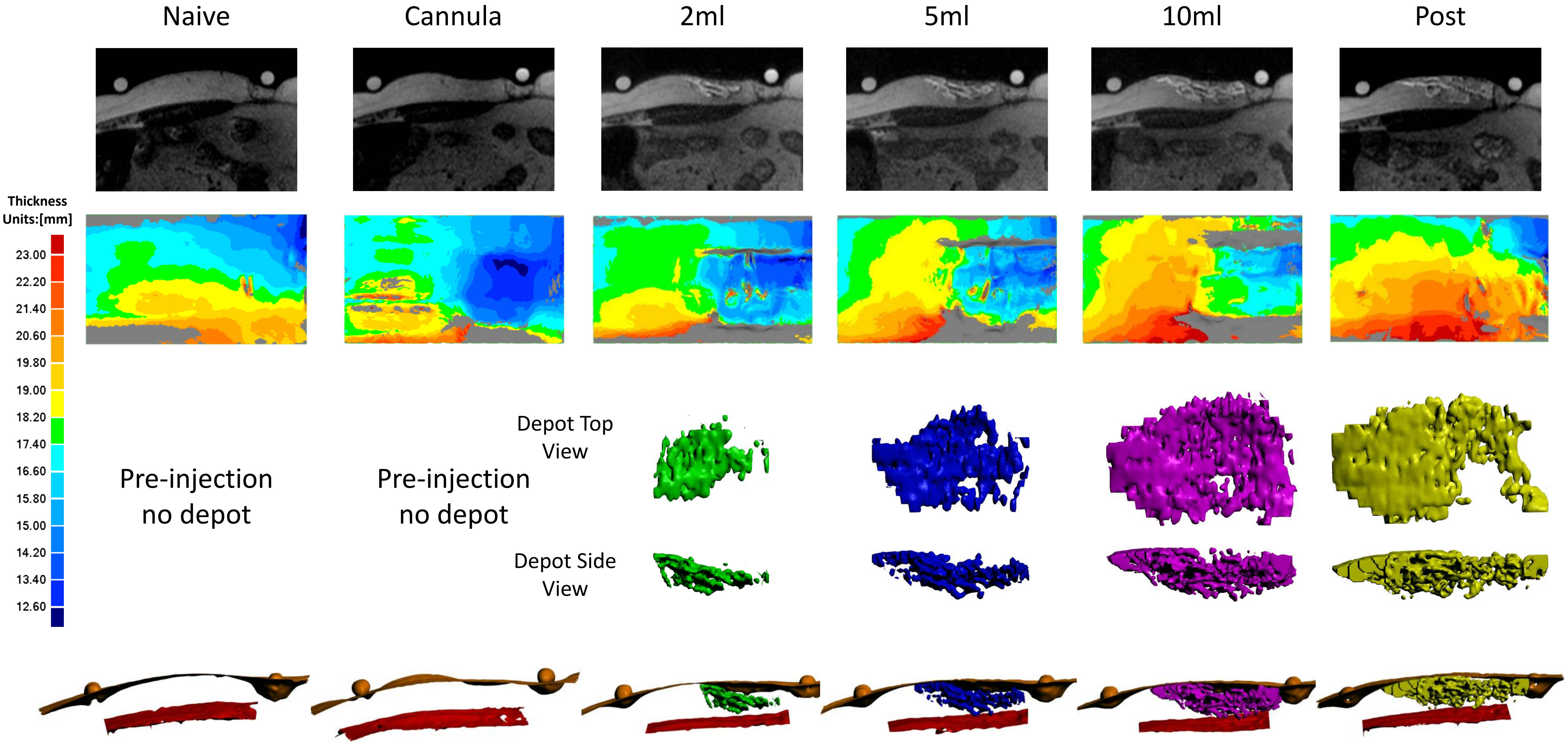

009 Thigh Left

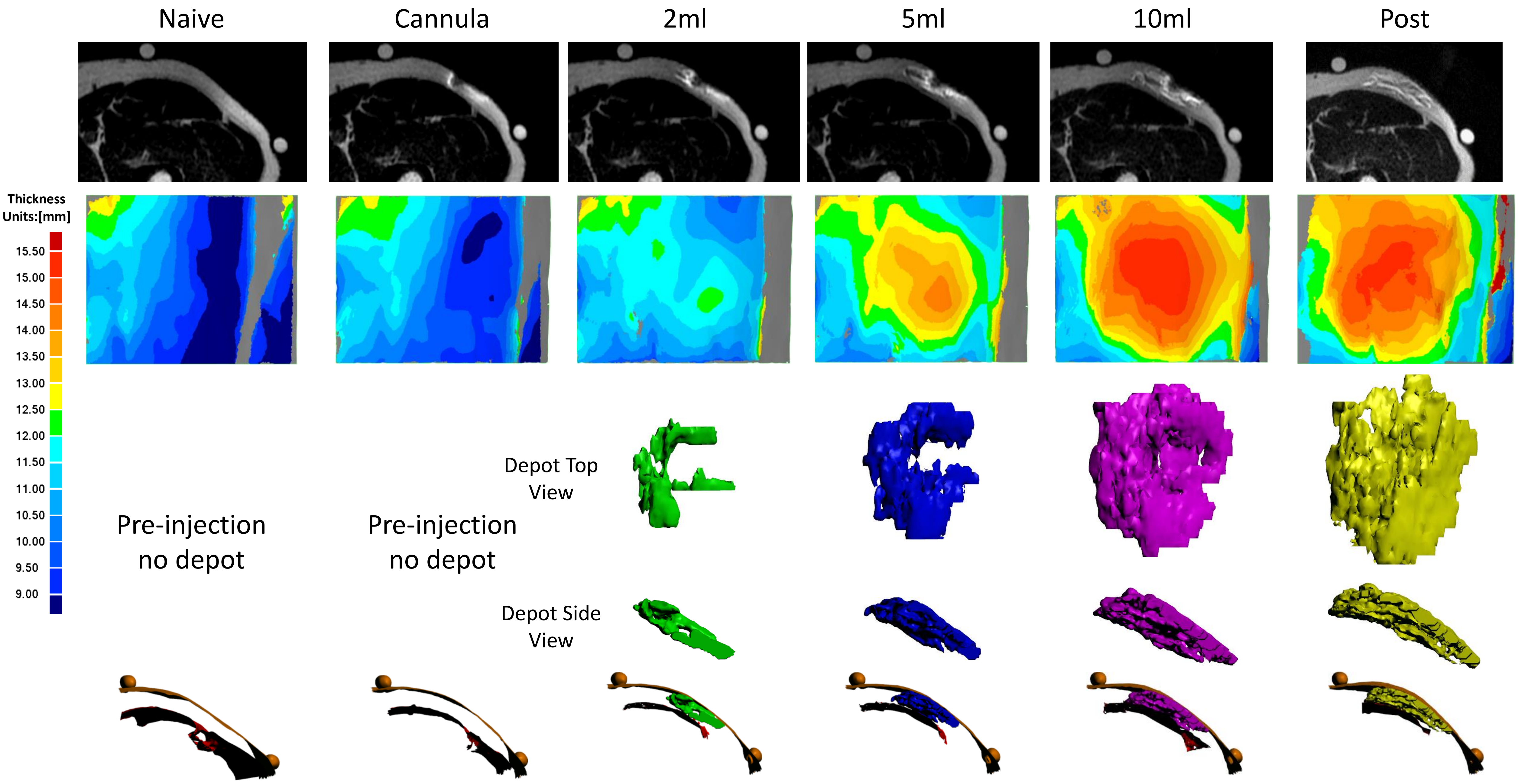

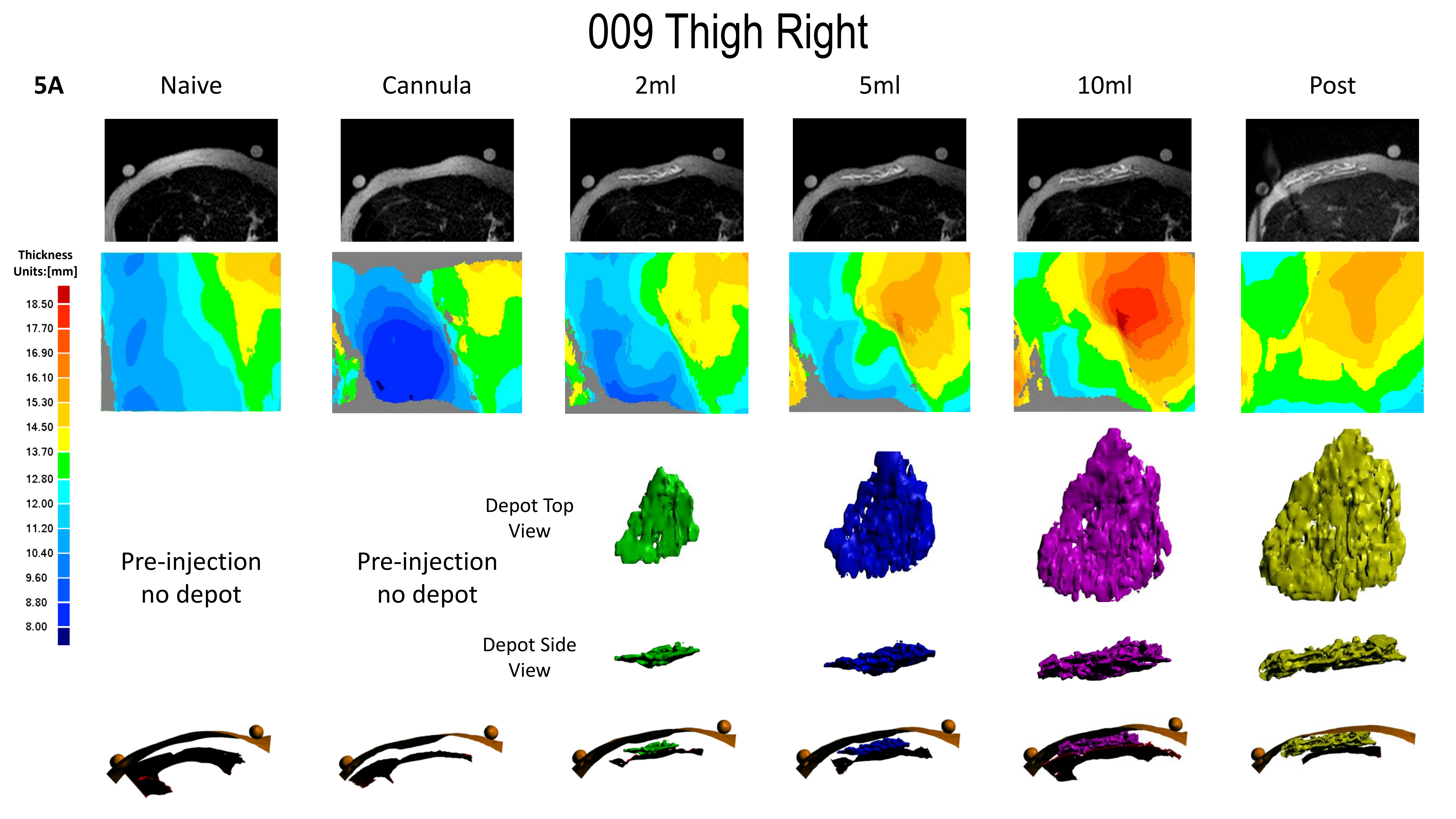
009 Thigh Right
5A
Naive
Cannula
2ml
5ml
10ml
Post
Thickness
Units:[mm]
18.50
17.70
16.90
16.10
15.30
14.50
13.70
12.80
12.00
11.20
10.40
9.60
8.80
8.00
Pre-injection
no depot
Pre-injection
no depot
Depot Top
View
Depot Side
View

010 Abdomen Left

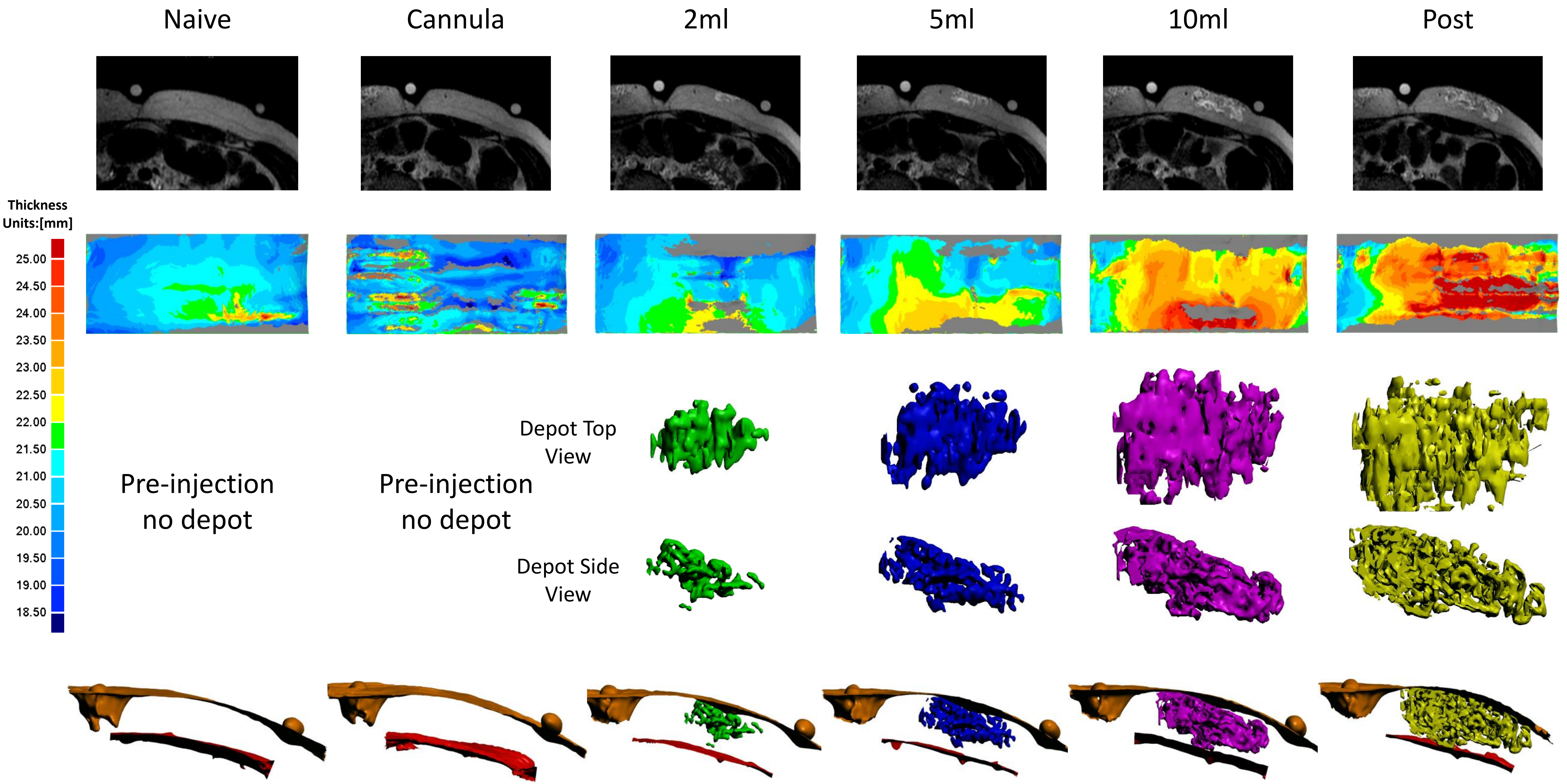

010 Abdomen Right

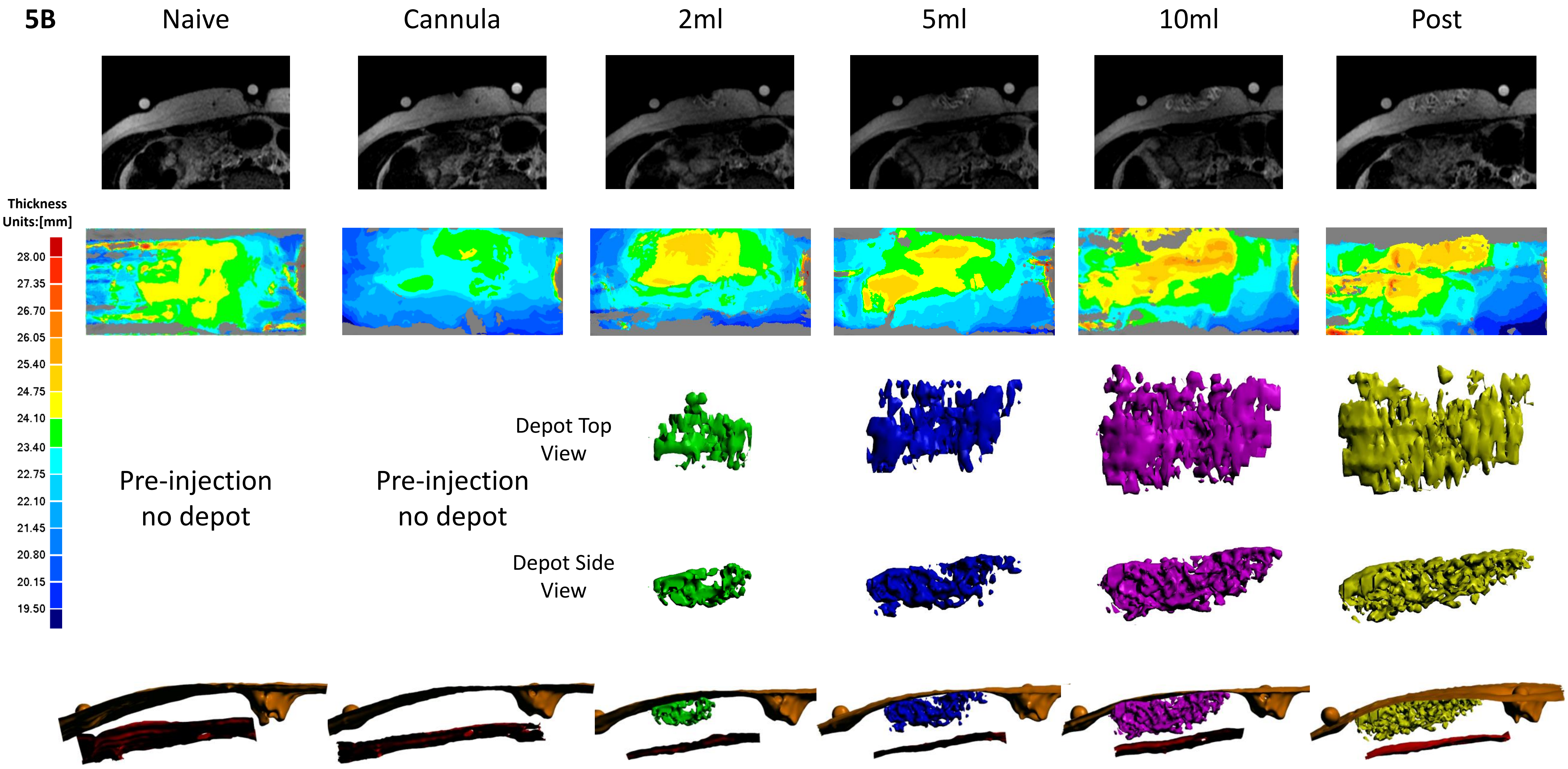

010 Arm Left

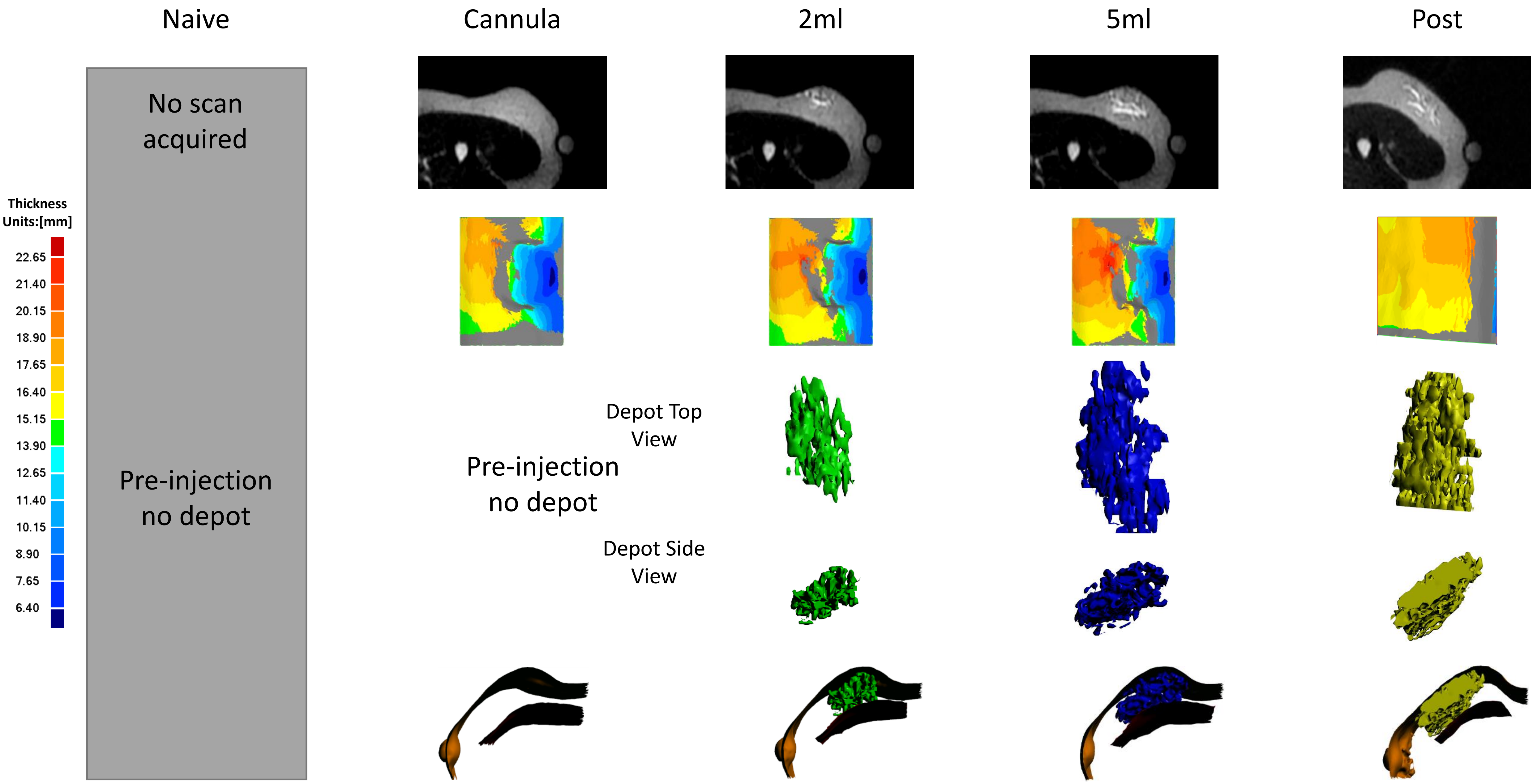
Naive
Cannula
2ml
5ml
Post
No scan acquired
Thickness Units:[mm]
22.65
21.40
20.15
18.90
17.65
16.40
15.15
13.90
12.65
11.40
10.15
8.90
7.65
6.40
Pre-injection no depot
Depot Top View
Pre-injection no depot
Depot Side View

010 Thigh Left

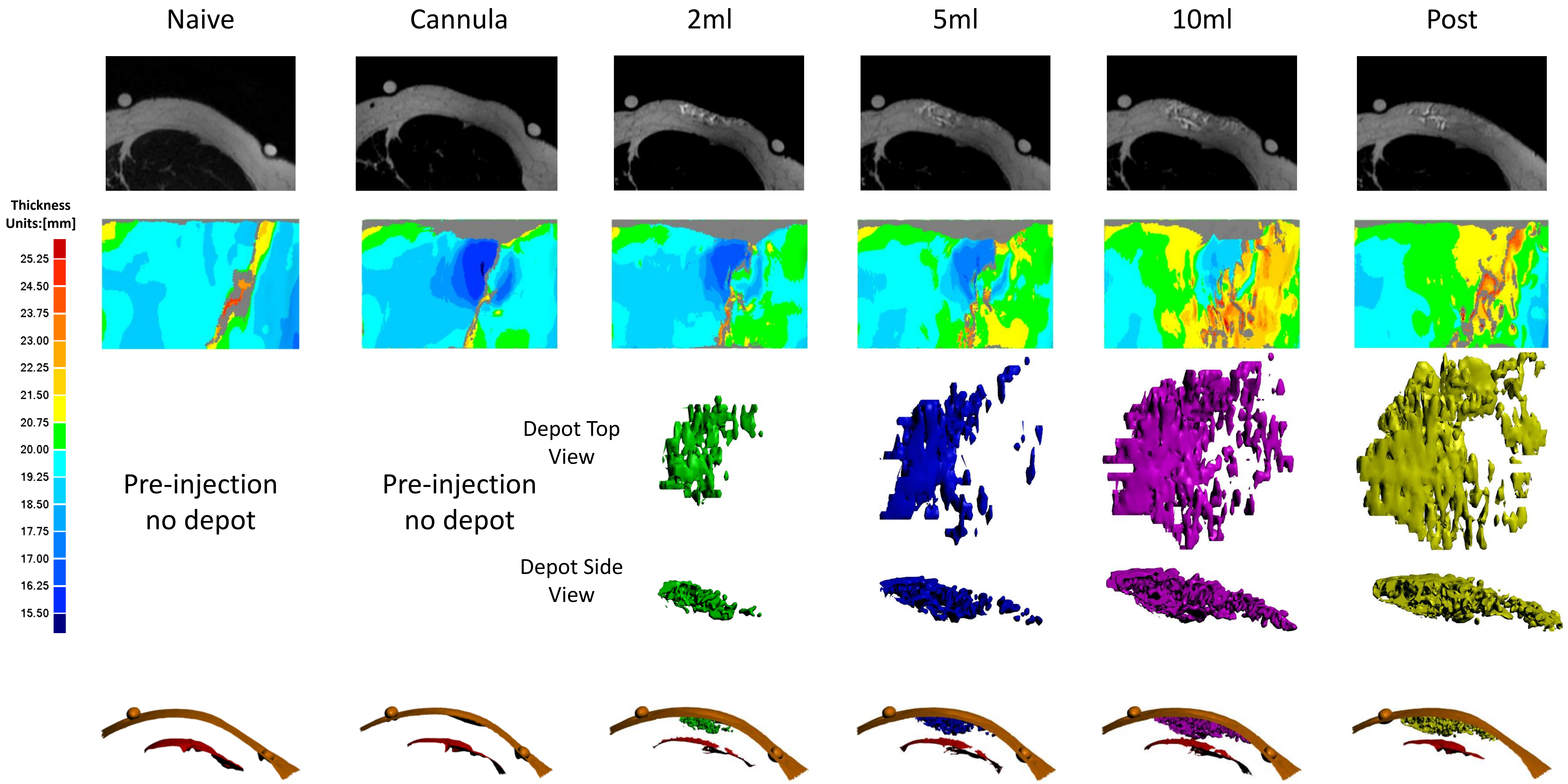

010 Thigh Right

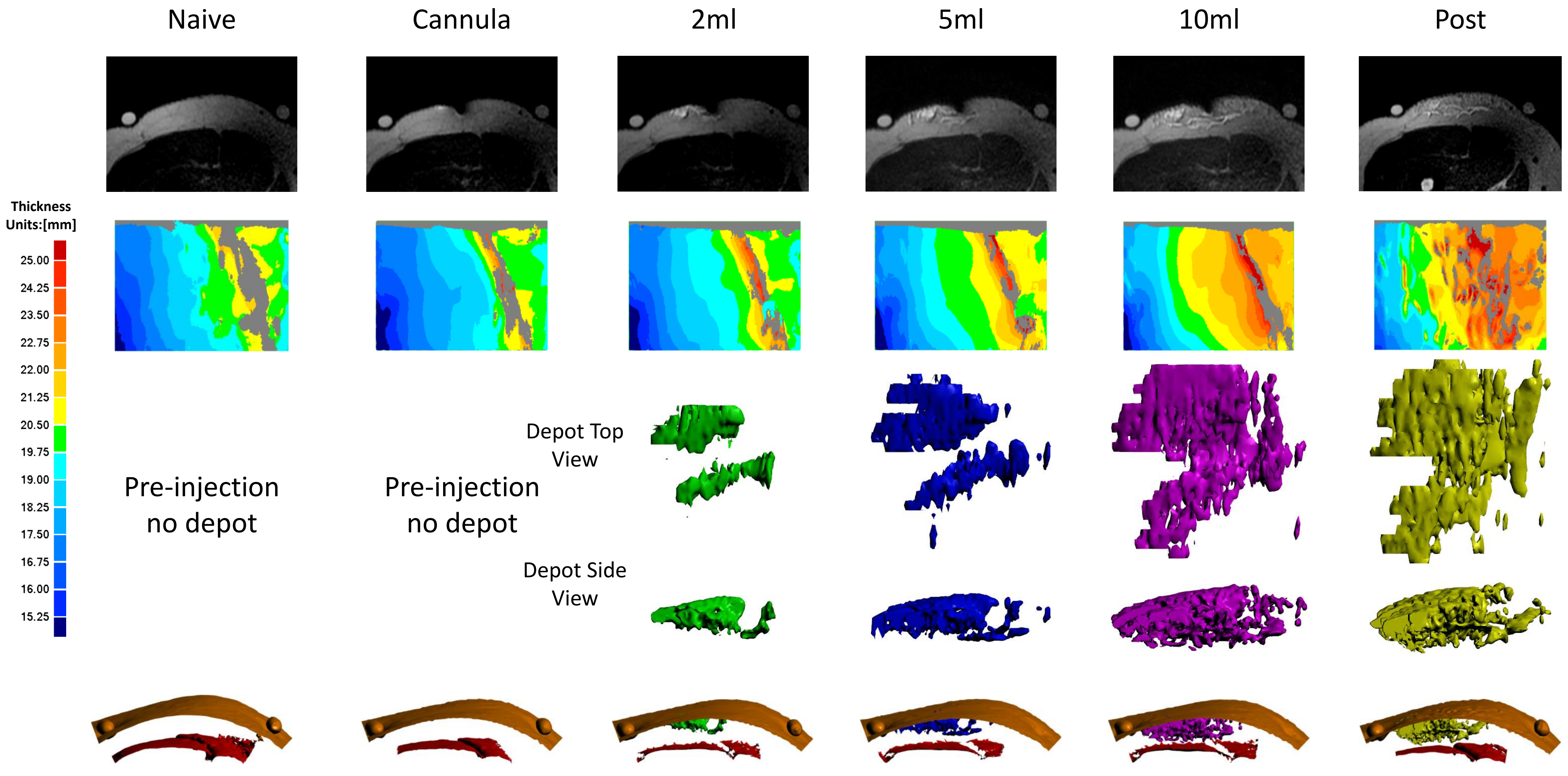

011 Abdomen Left

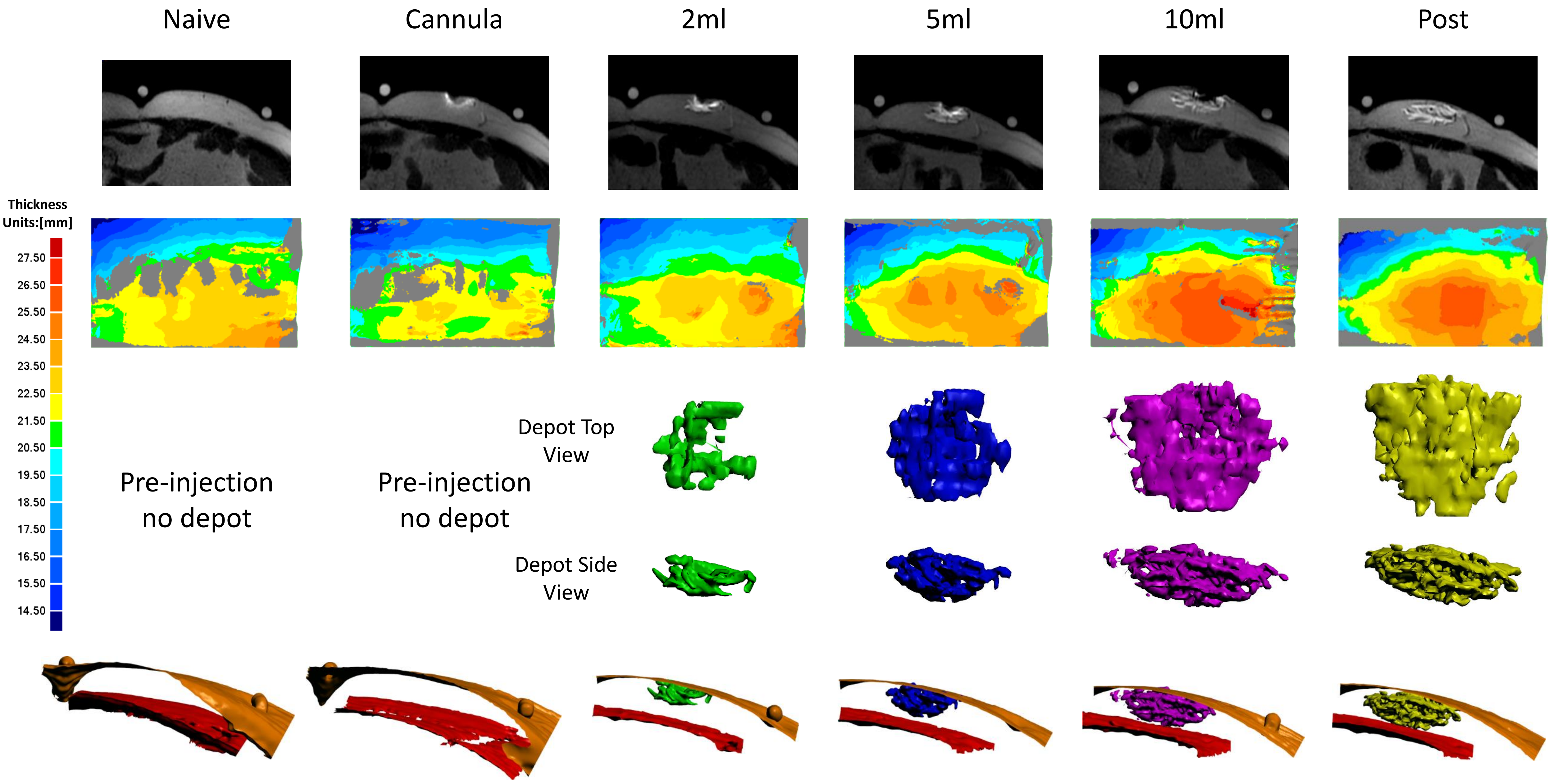

011 Abdomen Right

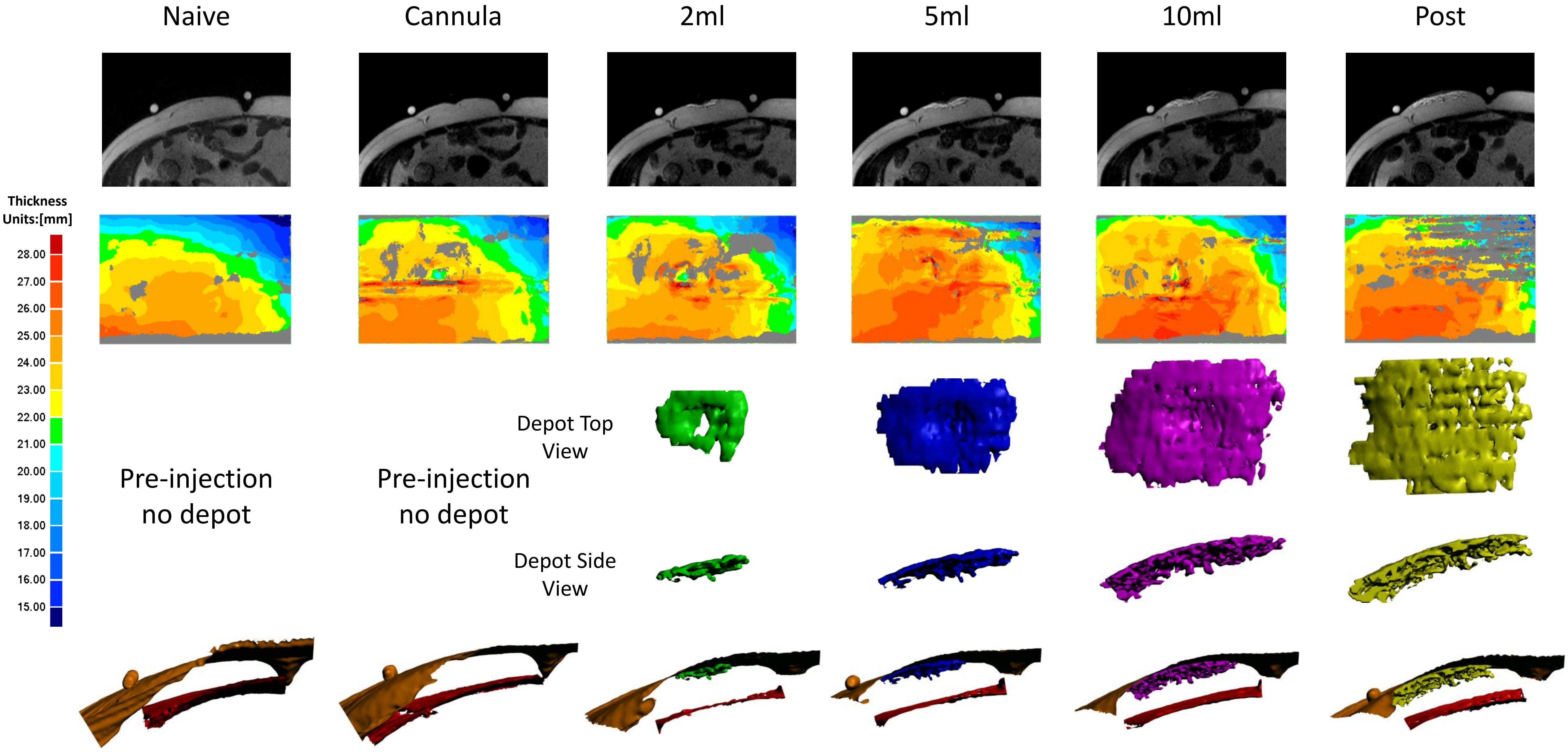

011 Arm Right

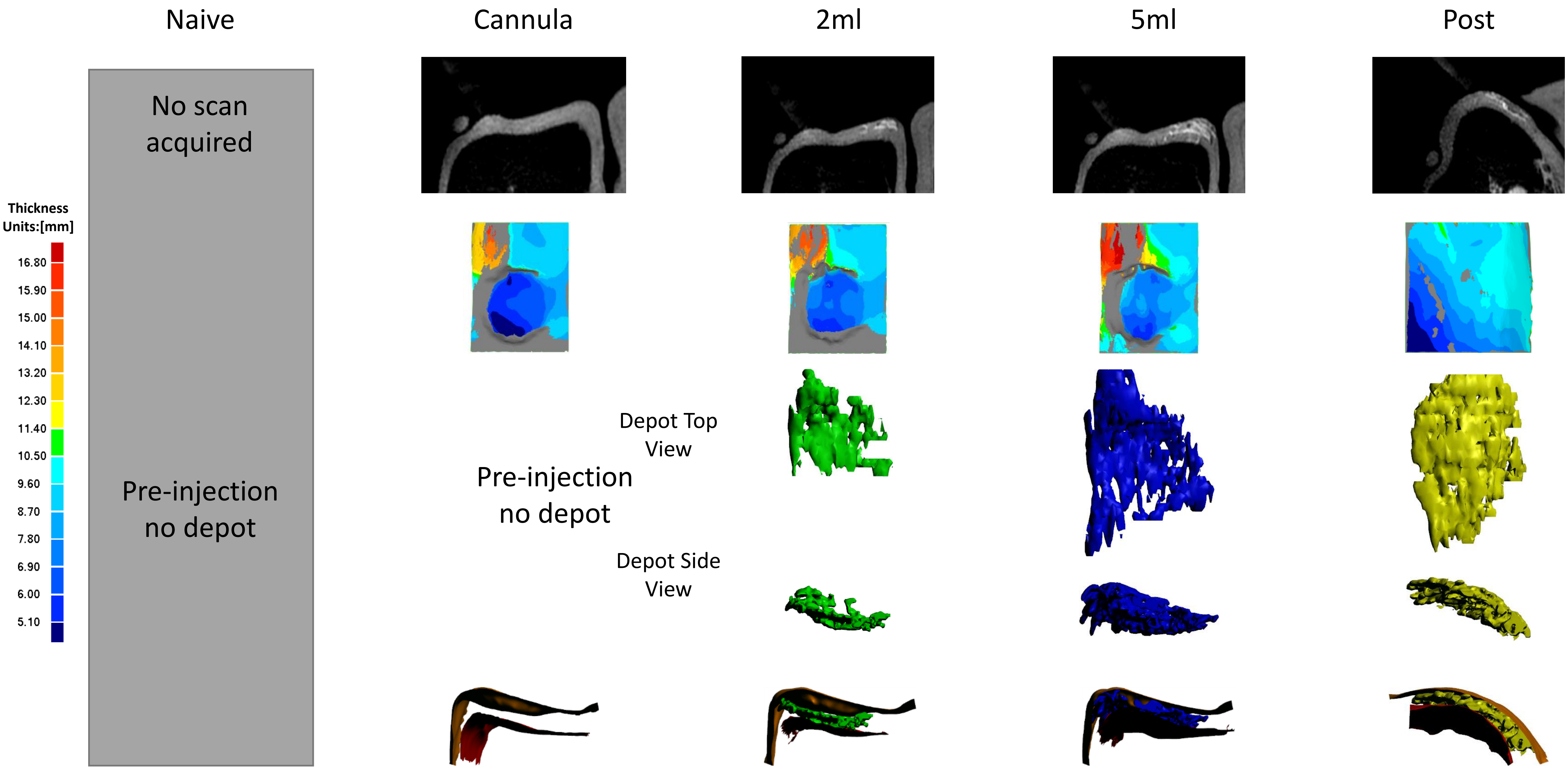

011 Thigh Left

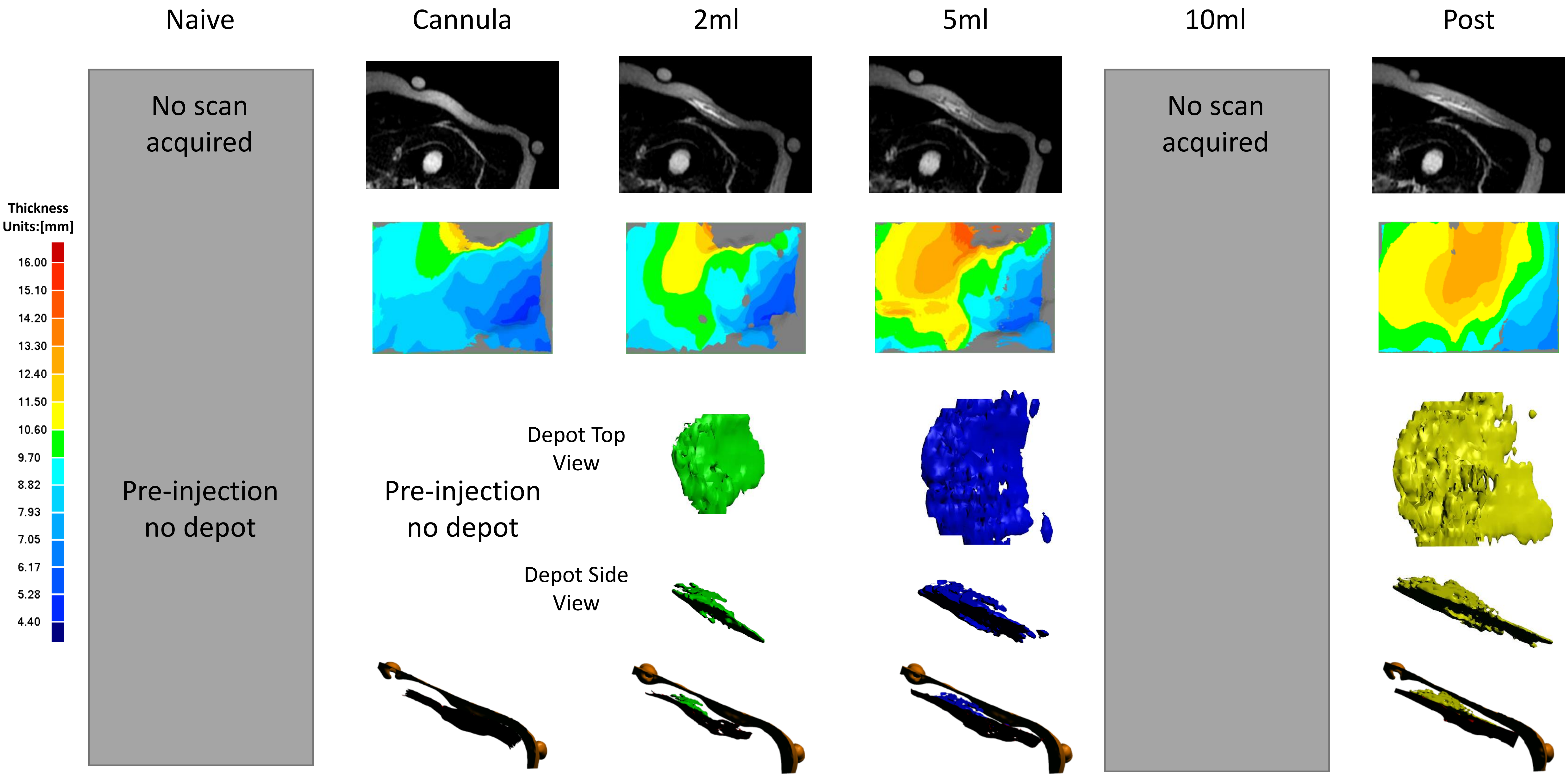

011 Thigh Right

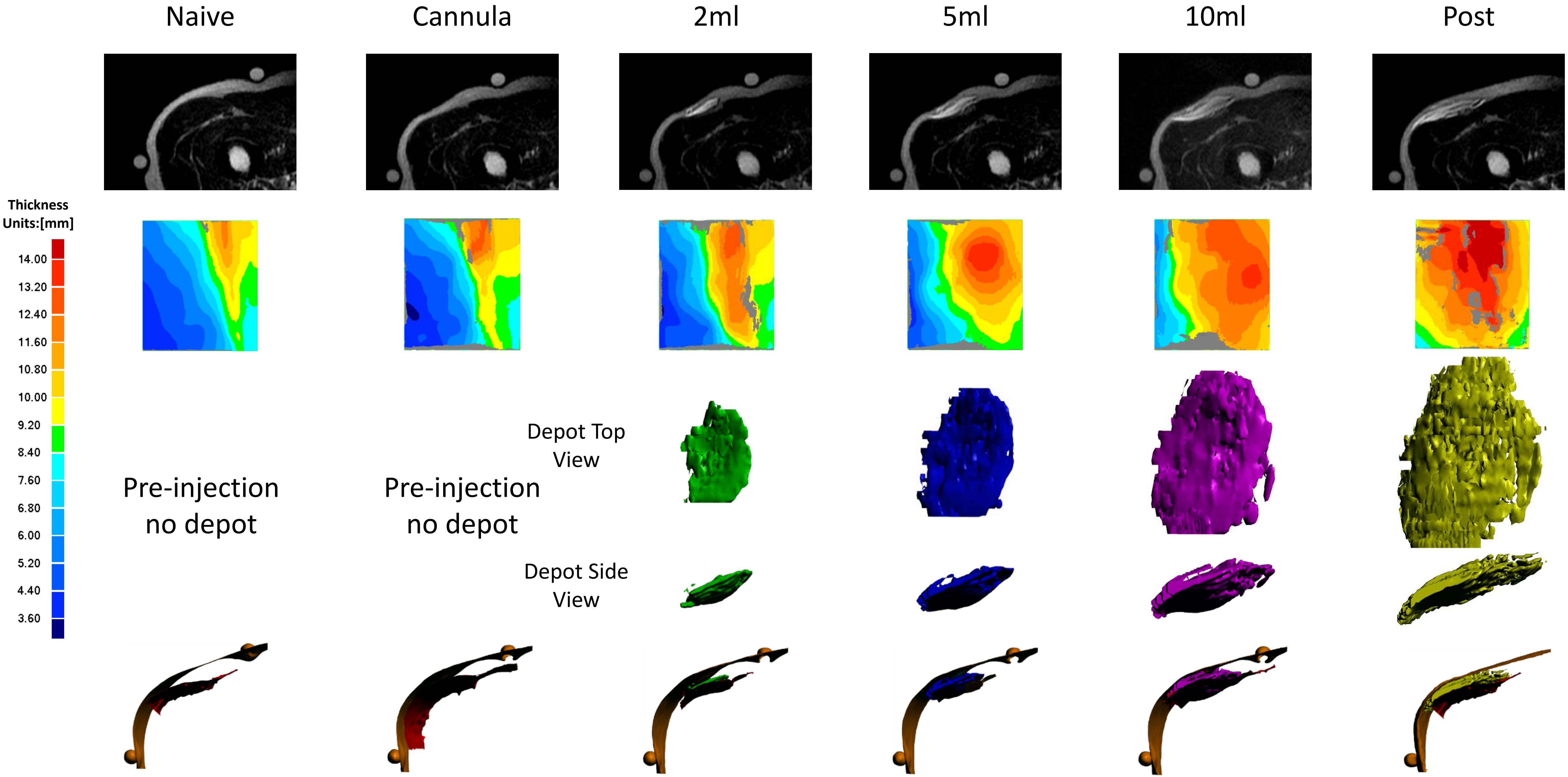

012 Abdomen Left

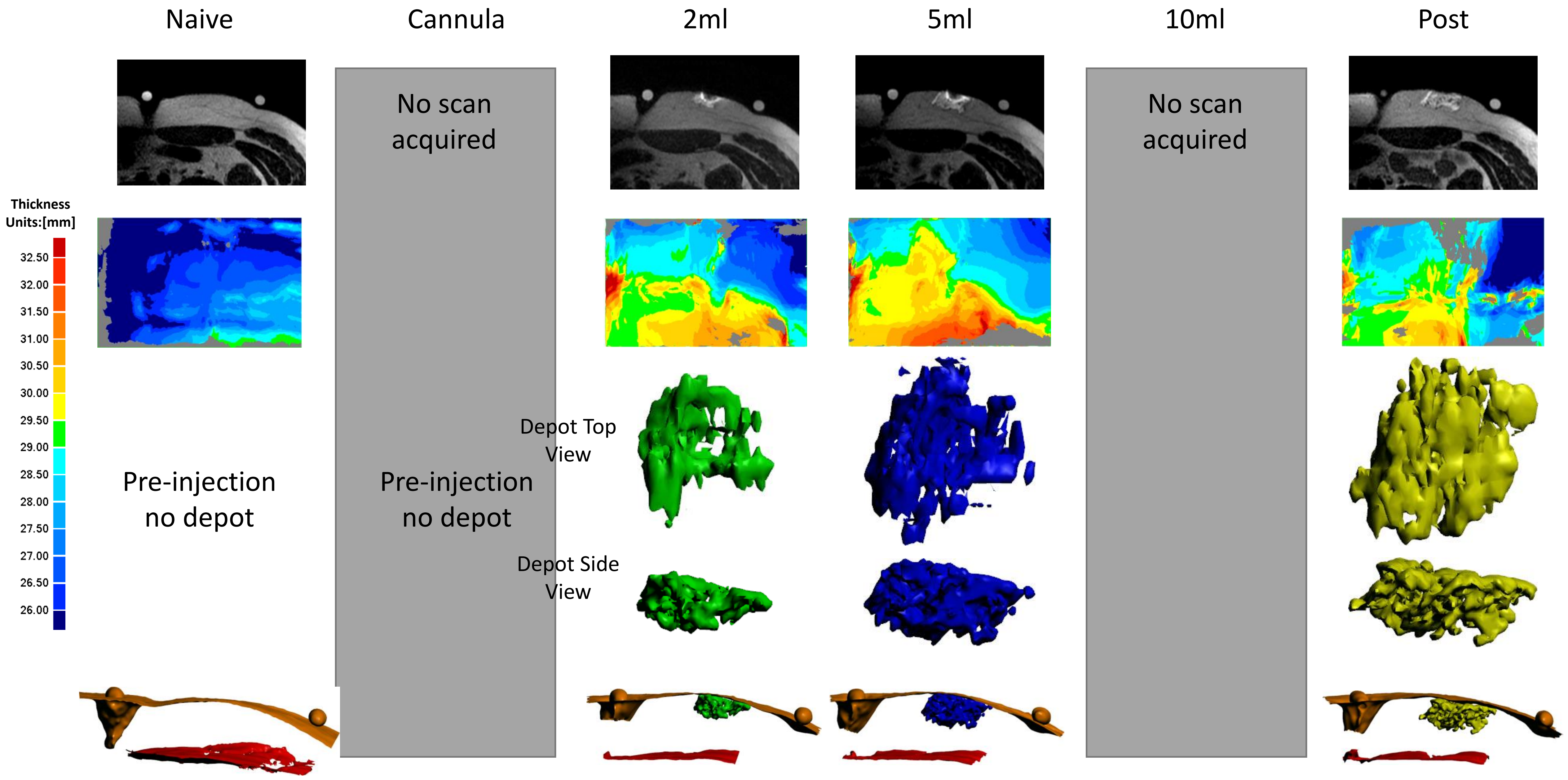

012 Abdomen Right

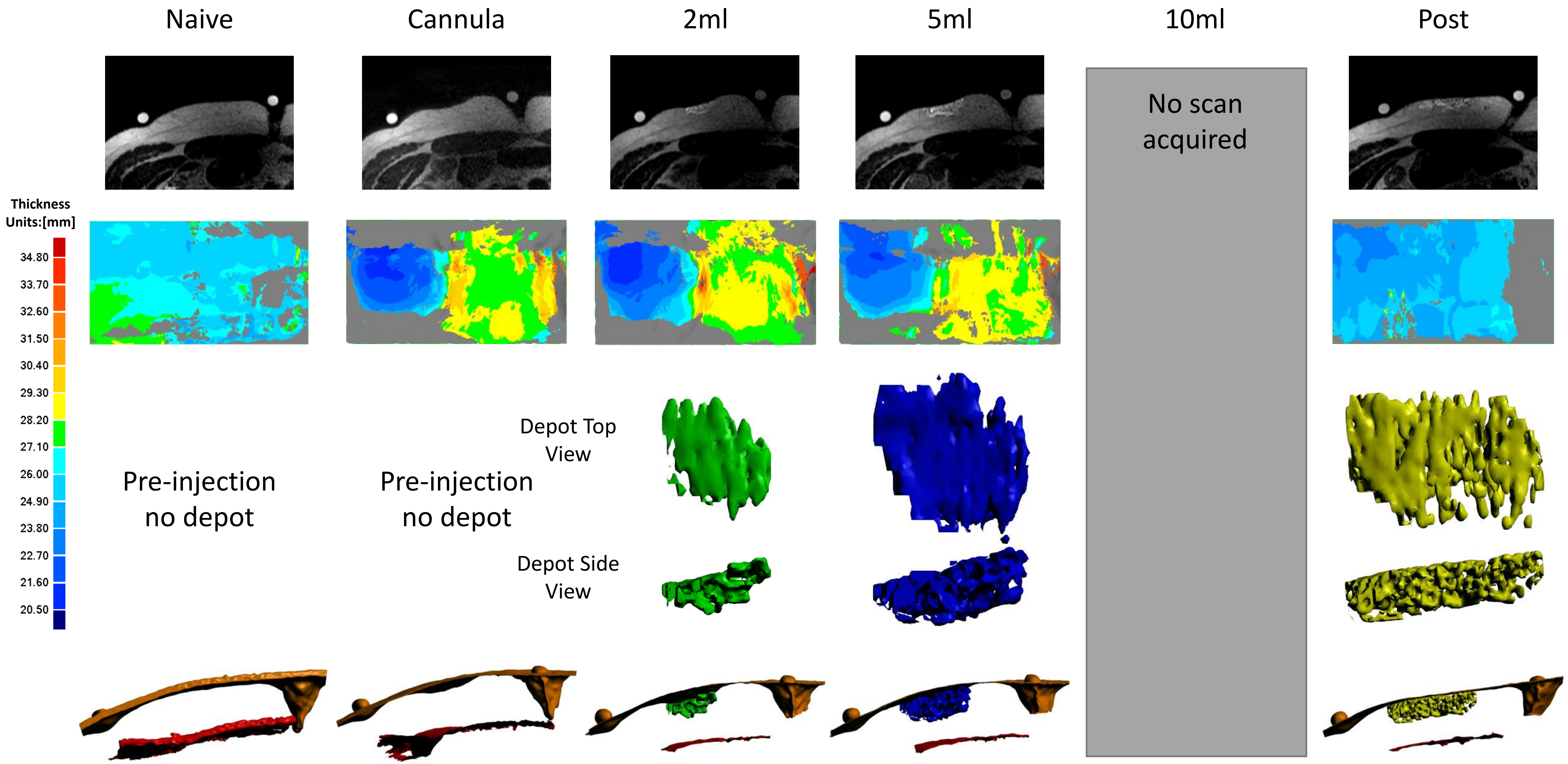

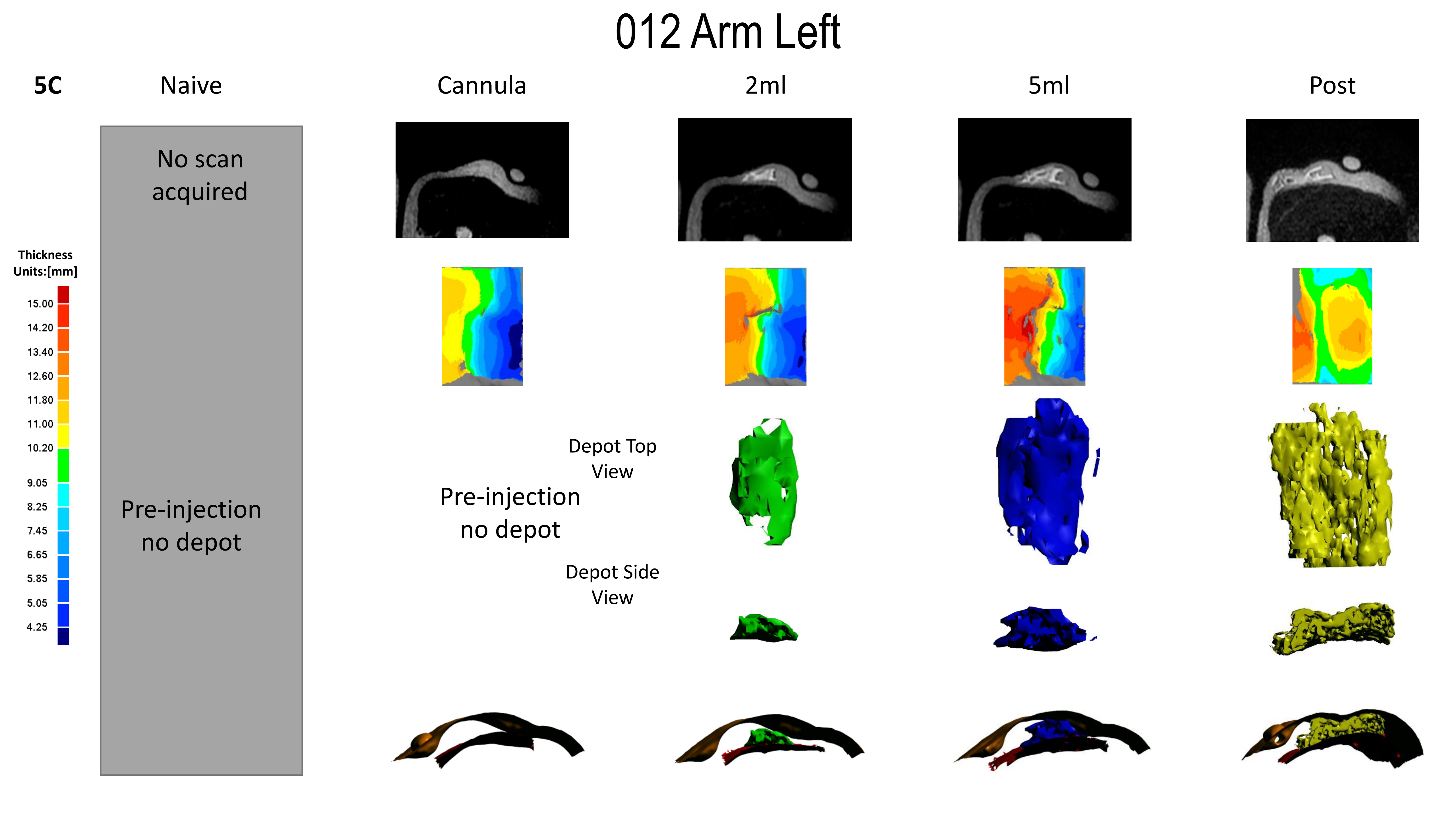

012 Arm Left
5C
Naive
Cannula
2ml
5ml
Post
No scan acquired
Thickness Units:[mm]
15.00
14.20
13.40
12.60
11.80
11.00
10.20
9.05
8.25
7.45
6.65
5.85
5.05
4.25
Depot Top View
Pre-injection no depot
Pre-injection no depot
Depot Side View

012 Thigh Left

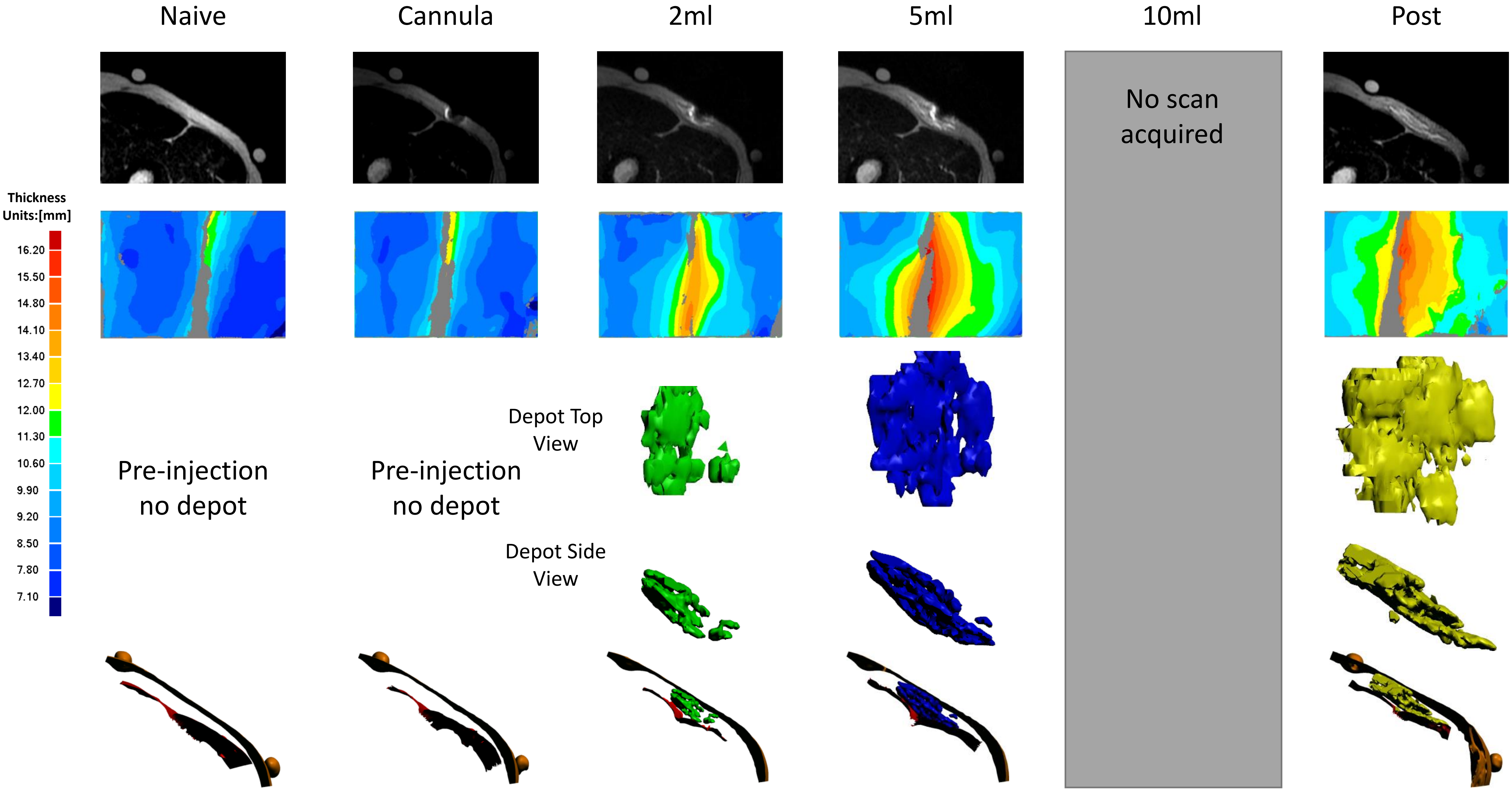

012 Thigh Right

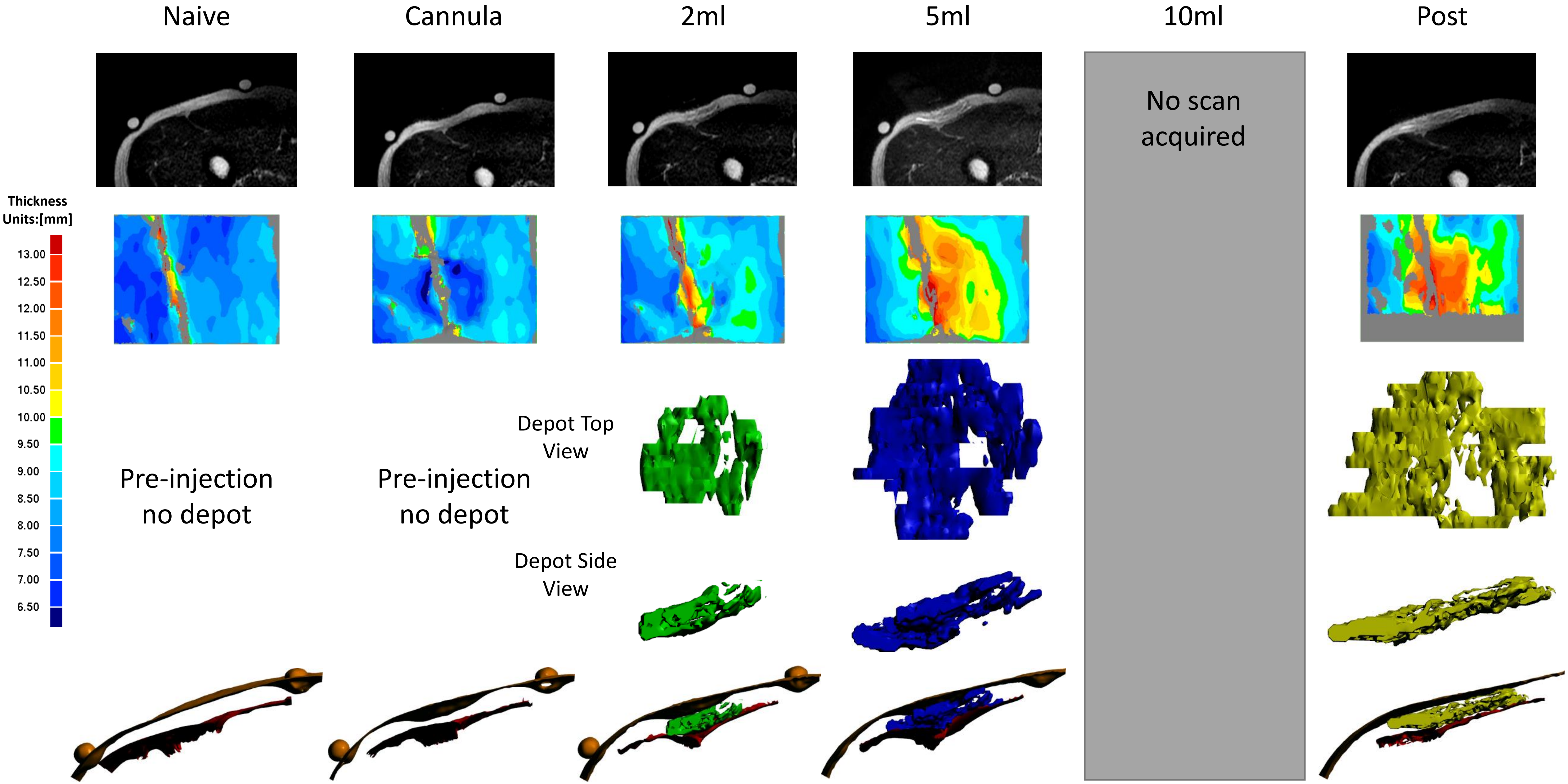

Supplement: Supplementary file 1 — Supplementary file1 (PDF 4.92 KB) [file 13346_2023_1318_MOESM1_ESM.pdf]
