# Supplementary material for: Imaging of large volume subcutaneous deposition using MRI: exploratory clinical study results
Source: Drug Deliv Transl Res. 2023 Mar 13;13(9):2353–66. doi: 10.1007/s13346-023-01318-7 (PMC10382358; doi:10.1007/s13346-023-01318-7)
Supplement: Supplementary file 2 — Supplementary file2 (PDF 159 KB) [file 13346_2023_1318_MOESM2_ESM.pdf]

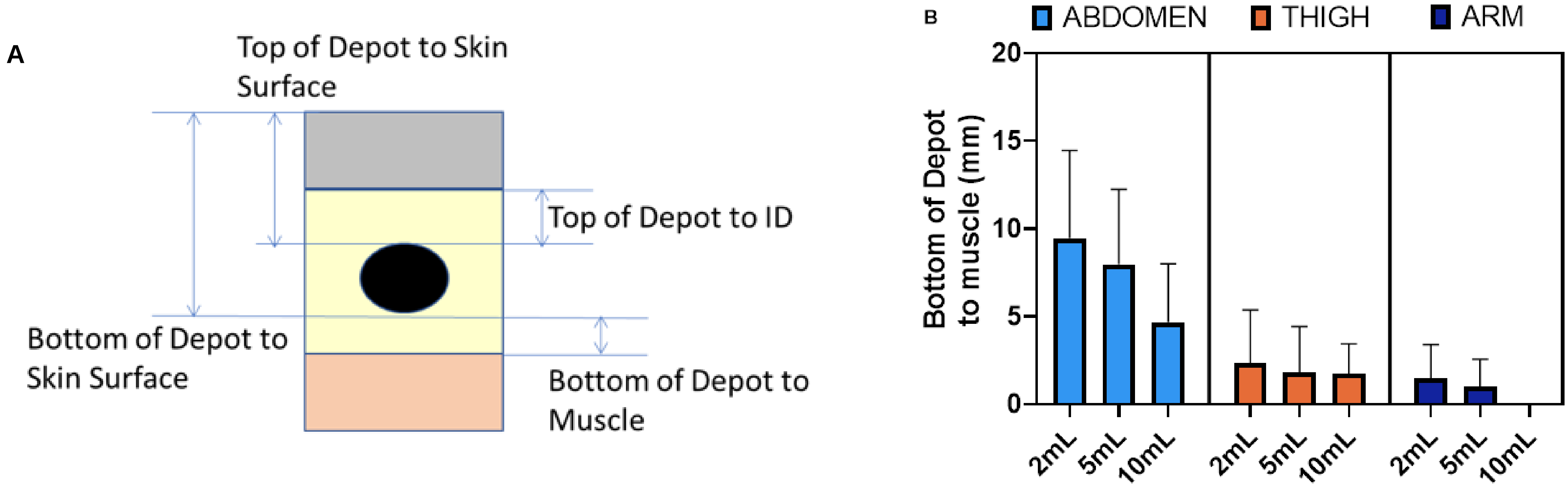

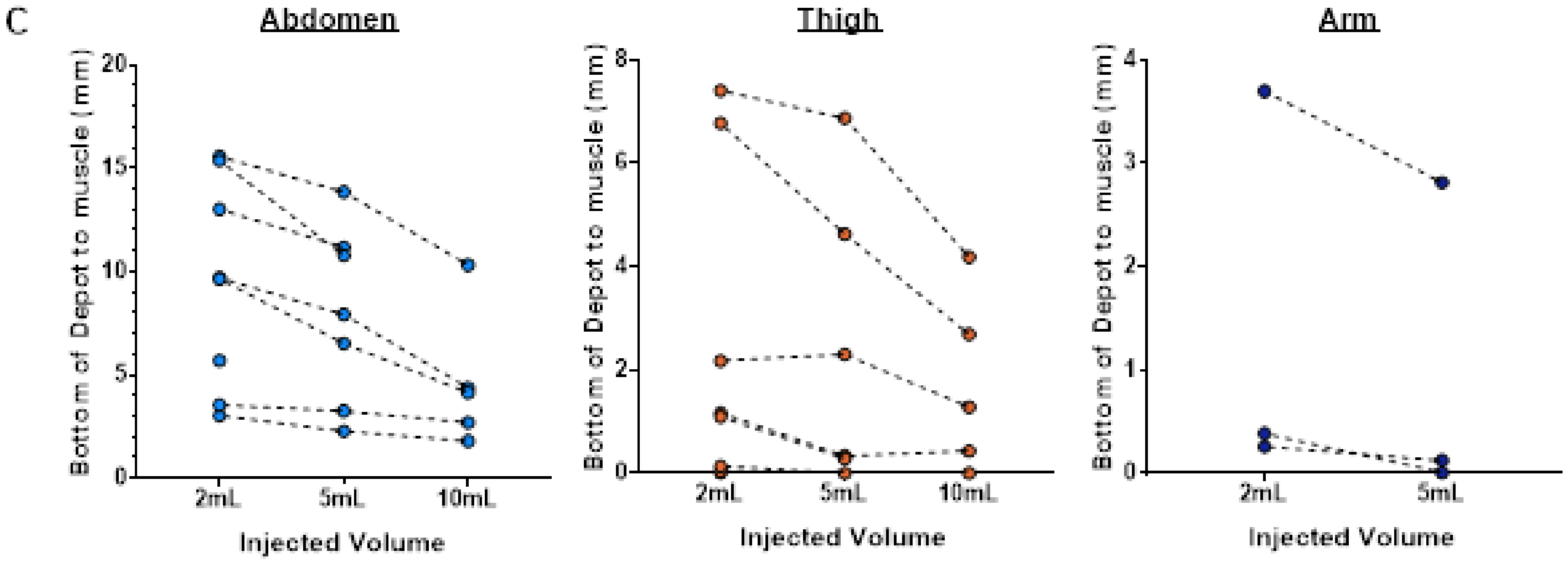

Supplemental Figure 2: A) Pictorial representation of distance measurements from depot to skin surface and ID/SC, SC/IM interface. B) Mean change in bottom depot border distance to muscle as a function of volume. C) Individual site changes in depot distance to muscle for each subject injection.

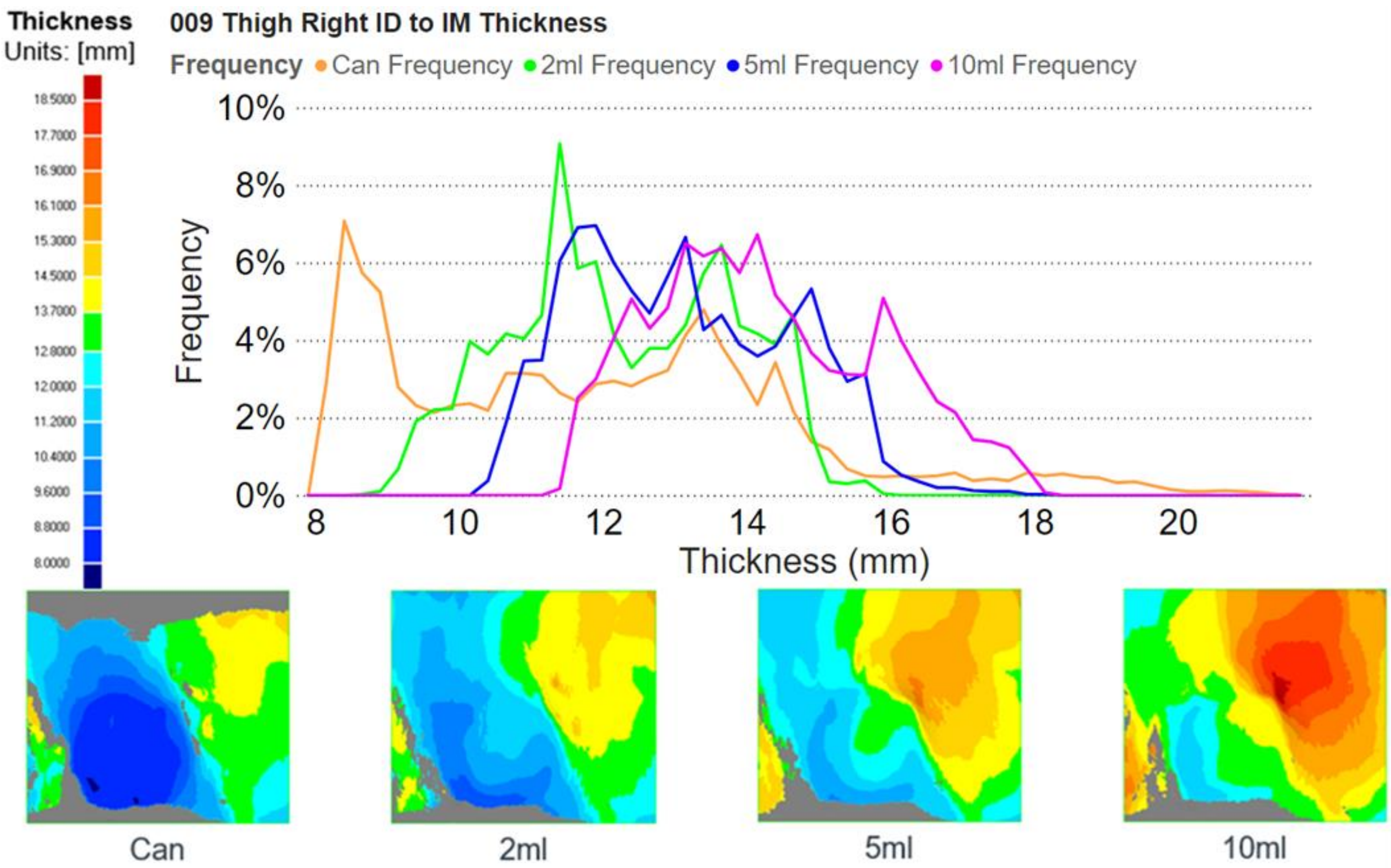

Supplemental Figure 3: Representative heat map images and histogram (Subject 009, Right thigh) characterizing the SC tissue thickness in the region of interest around the needle site, from cannulation through 10 mL delivery volume. Blue is the shortest distance (8.0 mm), dark red the longest distance (18.5 mm) and each color range represents an increase in SC thickness of 0.8mm. The change from blue to red demonstrates SC expansion as the tissue accommodates increasing depot volume. The histogram depicts the % frequency of each depth measurement across the region of interest shown in the heat map.
