# Supplementary material for: Imaging of large volume subcutaneous deposition using MRI: exploratory clinical study results
Source: Drug Deliv Transl Res. 2023 Mar 13;13(9):2353–66. doi: 10.1007/s13346-023-01318-7 (PMC10382358; doi:10.1007/s13346-023-01318-7)
Supplement: Supplementary file 3 — Supplementary file3 (PDF 90.2 KB) [file 13346_2023_1318_MOESM3_ESM.pdf]

Supplemental Tables

Supplemental Table 1: Descriptive Statistics for Projected Principal X-Y and Z Axes (mm), by injection site and target delivery volume

Site and Injection Volume	Principal Axes	n	Mean (mm)	SE mean	SD	Median	Min	Max
**Abdomen 2mL**	X	8	33.9	1.81	5.11	34.2	26.6	40.7
	Y	8	28.4	1.33	3.77	29.5	21.4	33.4
	Z	8	12.9	0.98	2.78	12.8	8.96	18.2
**Abdomen 5mL**	X	7	45	3.05	8.08	42.4	34.3	55.2
	Y	7	35.7	1.52	4.02	37.6	29.5	40.6
	Z	7	16.4	0.95	2.52	17.5	11.7	19.3
**Abdomen 5mL Post**	X	2	42.5	5.27	7.45	42.5	37.3	47.8
	Y	2	35.4	1.93	2.73	35.4	33.4	37.3
	Z	2	14.3	1.91	2.7	14.3	12.3	16.2
**Abdomen 10mL**	X	5	62.1	3.47	7.77	61	51.9	72.6
	Y	5	43.5	1.84	4.12	42.8	38.6	48.8
	Z	5	20.1	0.94	2.09	20.3	16.9	22.6
**Abdomen 10mL Post**	X	5	64	4.11	9.2	62.1	51.7	74.8
	Y	5	44.5	2.3	5.14	45	38.7	51.7
	Z	5	19.7	1.31	2.92	18.9	17.8	24.9
**Thigh 2mL**	X	8	33.8	1.48	4.18	34.6	27.5	38.3
	Y	8	34.5	1.46	4.12	35.6	27.3	39.1
	Z	8	10.3	0.43	1.21	10.2	8.8	12.6
**Thigh 5mL**	X	8	45.9	2.93	8.29	44.8	35.2	59.1
	Y	8	49.6	2.86	8.1	48.2	39.1	65.9
	Z	8	12.9	0.36	1.03	12.8	11.4	14.4
**Thigh 5mL Post**	X	3	52.8	4.12	7.13	56.2	44.6	57.7
	Y	3	44.5	3.86	6.69	42.1	39.3	52.1
	Z	3	11.3	1.12	1.94	10.8	9.58	13.4
**Thigh 10mL**	X	5	55.4	4.27	9.54	53.3	44.6	69
	Y	5	62.6	2.68	5.99	60.6	57.1	69.1
	Z	5	17.2	0.47	1.06	17.6	15.3	17.9
**Thigh 10mL Post**	X	5	58.2	2.73	6.1	55.6	52.7	68.3
	Y	5	66.3	1.31	2.93	66.2	62.9	69.5
	Z	5	15.9	0.99	2.2	17.2	13.3	17.7
**Arm 2mL**	X	3	26	4.66	8.07	22.3	20.4	35.2
	Y	3	39.5	1.96	3.4	38.7	36.6	43.2
	Z	3	12.9	1.15	1.99	12.1	11.5	15.2
**Arm 5mL**	X	3	35.1	4.63	8.02	30.5	30.4	44.4
	Y	3	57.5	3.67	6.36	58.8	50.6	63.1
	Z	3	21.3	1.16	2.01	21	19.5	23.5
**Arm 5mL Post**	X	3	38.9	1.89	3.27	39.1	35.6	42.1
	Y	3	53.3	3.75	6.5	51.5	47.9	60.5
	Z	3	15.8	1.88	3.25	14.5	13.4	19.5

Supplemental Table 2: Descriptive Statistics of depot reconstruction orthographic projected area (mm2) and final segmented volume (mL) by injection site and target delivery volume.

Site and Injection Volume	Metric	n	Mean	SE mean	SD	Median	Min	Max
**Abdomen 2mL**	Proj. Area	8	519	31.9	90.3	499	416	678
	Seg. Volume	8	1.7	0.08	0.23	1.68	1.33	2.14
**Abdomen 5mL**	Proj. Area	7	1054	109	289	1049	703	1562
	Seg. Volume	7	4.35	0.24	0.63	4.38	3.49	5.32
**Abdomen 5mL Post**	Proj. Area	2	1032	151	213	1032	881	1182
	Seg. Volume	2	4.43	0.46	0.65	4.43	3.97	4.89
**Abdomen 10mL**	Proj. Area	5	1901	243	542	1614	1380	2713
	Seg. Volume	5	8.88	0.17	0.37	8.84	8.41	9.33
**Abdomen 10mL Post**	Proj. Area	5	1985	252	563	1750	1510	2902
	Seg. Volume	5	9.03	0.447	1	8.63	8.25	10.6
**Thigh 2mL**	Proj. Area	8	643	48.5	137	627	467	856
	Seg. Volume	8	1.92	0.13	0.37	1.84	1.46	2.73
**Thigh 5mL**	Proj. Area	8	1288	75.9	215	1243	1022	1671
	Seg. Volume	8	4.49	0.17	0.49	4.59	3.4	4.99
**Thigh 5mL Post**	Proj. Area	3	1622	269	467	1403	1306	2158
	Seg. Volume	3	4.41	0.69	1.2	3.99	3.48	5.76
**Thigh 10mL**	Proj. Area	5	2144	81.3	182	2036	1991	2370
	Seg. Volume	5	9.62	0.15	0.34	9.54	9.13	9.99
**Thigh_10mL Post**	Proj. Area	5	2503	129	289	2662	2085	2771
	Seg. Volume	5	10	0.20	0.44	10.2	9.33	10.4
**Arm 2mL**	Proj. Area	3	630	56	97.1	605	548	737
	Seg. Volume	3	1.83	0.04	0.06	1.82	1.78	1.9
**Arm 5mL**	Proj. Area	3	1250	145	251	1161	1055	1533
	Seg. Volume	3	4.64	0.16	0.28	4.75	4.33	4.86
**Arm 5mL Post**	Proj. Area	3	1489	155	268	1547	1196	1724
	Seg. Volume	3	5.26	0.07	0.13	5.3	5.12	5.36

Supplemental Table 3: Compiled Summary Data for SC Thickness Measurements per Injection Site and Volume

Site	Sequence	n	Mean	SE mean	SD	Median	Min	Max
**Abdomen**	Cannula Placed	7*	19.0	1.5	4.0	20.6	12.6	22.2
	2 mL	8	20.0	1.6	4.4	21.1	12.8	26.4
	5 mL	7	21.5	1.5	3.9	22.0	14.0	27.0
	10 mL	5	20.3	1.7	3.8	21.9	13.5	22.4
**Thigh**	Cannula Placed	8	10.2	1.5	4.3	8.9	4.2	17.2
	2 mL	8	11.3	1.6	4.4	11.0	4.1	17.8
	5 mL	8	12.2	1.6	4.3	12.2	4.6	18.3
	10 mL	5	14.3	2.5	5.5	14.5	5.3	18.8
**Arm**	Cannula Placed	3	8.8	1.3	2.2	9.6	6.4	10.5
	2 mL	3	9.4	1.3	2.2	10.1	6.8	11.1
	5 mL	3	9.9	1.4	2.5	11.1	7.1	11.7

* Cannula placement image not obtained for 012 abdomen, therefore not included in measurements.

Supplemental Table 4: Compiled Summary Data for % Change in SC Thickness Compared to Cannula Placement for Each Injection Site and Volume

Site	Sequence	n	Mean	SE mean	SD	Median	Min	Max
**Abdomen**	Cannula Placed	7	NA	NA	NA	NA	NA	NA
	2 mL	7*	0.7	1.0	2.8	1.6	-5.2	3.2
	5 mL	6*	4.5	1.6	3.8	3.9	-0.2	11.3
	10 mL	5	5.2	1.5	3.3	6.9	0.8	8.1
**Thigh**	Cannula Placed	8	NA	NA	NA	NA	NA	NA
	2 mL	8	11.2	3.3	9.2	11.3	-1.7	26.1
	5 mL	8	21.3	4.5	12.8	20.9	5.9	41.1
	10 mL	5	28.5	8.5	18.9	25.5	8.9	57.6
**Arm**	Cannula Placed	3	NA	NA	NA	NA	NA	NA
	2 mL	3	5.9	0.9	1.6	5.4	4.7	7.7
	5 mL	3	12.2	1.3	2.3	11.3	10.5	14.8

* Cannula placement image not obtained for 012 abdomen, therefore % change could not be calculated.
